# Dolichol kinases from yeast, nematode and human can replace each other and exchange their domains creating active chimeric enzymes in yeast

**DOI:** 10.1371/journal.pone.0313330

**Published:** 2024-11-07

**Authors:** Danguole Ziogiene, Andrius Burdulis, Albertas Timinskas, Ruta Zinkeviciute, Emilija Vasiliunaite, Milda Norkiene, Alma Gedvilaite

**Affiliations:** Institute of Biotechnology, Life Sciences Center, Vilnius University, Vilnius, Lithuania; CNR, ITALY

## Abstract

Protein glycosylation is a fundamental modification crucial for numerous intra- and extracellular functions in all eukaryotes. The phosphorylated dolichol (Dol-P) is utilized in N-linked protein glycosylation and other glycosylation pathways. Dolichol kinase (DK) plays a key role in catalyzing the phosphorylation of dolichol. The glycosylation patterns in the *Kluyveromyces lactis* DK mutant revealed that the yeast well tolerated a minor deficiency in Dol-P by adjusting protein glycosylation. Comparative analysis of sequences of DK homologs from different species of eukaryotes, archaea and bacteria and AlphaFold3 structural model studies, allowed us to predict that DK is most likely composed of two structural/functional domains. The activity of predicted *K*. *lactis* DK C-terminal domain expressed from the single copy in the chromosome was not sufficient to keep protein glycosylation level necessary for survival of *K*. *lactis*. However, the glycosylation level was partially restored by additionally provided and overexpressed N- or C-terminal domain. Moreover, co-expression of the individual N-and C-terminal domains restored the glycosylation of vacuolar carboxypeptidase Y in both *K*. *lactis* and *Saccharomyces cerevisiae*. Despite the differences in length and non-homologous sequences of the N-terminal domains the human and nematode *Caenorhabditis elegans* DKs successfully complemented DK functions in both yeast species. Additionally, the N-terminal domains of *K*. *lactis* and *C*. *elegans* DK could functionally substitute for one another, creating active chimeric enzymes. Our results suggest that while the C-terminal domain remains crucial for DK activity, the N-terminal domain may serve not only as a structural domain but also as a possible regulator of DK activity.

## Introduction

Protein glycosylation is a fundamental modification process crucial for protein folding, quality control, sorting events, and numerous intra- and extracellular functions within the secretory pathway in both lower and higher eukaryotes [1–4]. The initial stages of the glycosylation process in the endoplasmic reticulum (ER) demand sufficient amounts of the phosphorylated isoprenoid lipid dolichol (Dol-P), which plays a pivotal role in various glycosylation pathways [5]. Dol-P serves as the carrier lipid for synthesis of oligosaccharide chains necessary for the assembly of N-linked glycoproteins. Additionally, it is utilized for the shuttling of Dol-P-linked sugars (Dol-P-mannose and Dol-P-glucose) serving not only as substrates for protein N-glycosylation but also for protein O-mannosylation and the biosynthesis of GPI-anchored proteins [5–9]. An adequate cellular concentration of Dol-P is maintained by dolichol kinase (DK), an ER membrane protein, which facilitates the transfer of the phosphoryl group from CTP to dolichol. DK plays a pivotal role in catalyzing the final step of the *de novo* pathway for the Dol-P formation in the ER of both yeast and animal cells [2,10,11]. DK may also play a role in the “reactivation” of extra-ER reserve pools of dolichol and in the recycling of the carrier lipid. This occurs when Dol-P is discharged during N-glycosylation reactions on the luminal surface, and the free dolichol diffuses back to the cytoplasmic side of the ER after dephosphorylation [10]. Consequently, by indirectly regulating the level of Dol-P in the ER through its synthesis, DK is involved in the regulation of protein glycosylation [5,10,11].

Recent studies have linked various human DK mutations to deficiencies in the N-linked glycosylation pathway, leading to inherited human diseases known as congenital disorders of glycosylation [12]. Furthermore, research has demonstrated the crucial role of DK in plant reproductive processes [13,14]. In zebrafish DK plays critical role in the spinal cord to regulate movement magnitude during the startle response and spontaneous swim movements [15]. *Saccharomyces cerevisiae* DK is encoded by an essential *SEC59* (SECretory) gene [16]. The studies of the conditional *sec59-1* mutant indicated that the reduced Dol-P levels in membrane of this mutant led to an impairment in protein glycosylation capability. Consequently, *sec59-1* yeast cells cultured at the restrictive temperature produced incompletely N-glycosylated and inactive proteins that accumulated in the ER. This accumulation resulted in the decreased secretion efficacy of vacuolar carboxypeptidase Y (CPY), invertase, and alpha-factor [16,17]. Furthermore, the *sec59-1* mutant exhibited deficiencies in O-linked glycosylation and GPI anchor synthesis, resulting in an abnormal cell wall composition and ultrastructure [18–20]. In contrast, similar glycosylation defects in the *Kluyveromyces lactis* conditional *klsec59* mutant WSS (G405S and I419S) were less severe and did not impact cell wall integrity [21]. Interestingly, although the secretion efficacy of invertase was slightly decreased in the WSS mutant, the secretion level of recombinant α-amylase was three times higher than in the wild type (WT) strain [21].

DK is characterized as a hydrophobic protein with its C-terminus exposed to the cytoplasmic face of the ER membrane. Previous predictions suggested the presence of up to 15 transmembrane domains in the DK sequence [22,23]. Although the experimental determination of DK’s structure has not been achieved, a recent prediction using AlphaFold technology has provided insights into its structural arrangement [24,25]. Structure-function studies on DK reveal that its C-terminus harbors a CTP-binding pocket and plays a crucial role in facilitating the transfer of the phosphoryl group from CTP to dolichol. Mutations affecting conserved residues within the C-terminus or the deletion of this region in *S*. *cerevisiae* or human DK led to a partial or complete loss of kinase activity [22].

In this study, we conducted a search for DK homologs and performed sequence alignments, which suggested that DK might consist of two distinct structural/functional domains. The presence of two structural domains was also predicted based on AlphaFold3 structural models of the *K*. *lactis* DK. Therefore, we separated predicted *K*. *lactis* DK domains and experimentally demonstrated that the overproduced C-terminal domain alone maintains dolichol phosphorylation level sufficient to sustain cell growth in *K*. *lactis* or restore mutated DK activity in *S*. *cerevisiae*. Furthermore, our experiments revealed that despite differences in length and the nonhomologous sequences of the N-terminal domains, these domains of *K*. *lactis* and *Caenorhabditis elegans* DK could functionally substitute for one another creating active chimeric DK enzymes. Additionally, the DK from either humans or nematodes, when expressed from a single chromosomal copy complemented DK functions in both yeast species *K*. *lactis* and *S*. *cerevisiae*.

## Materials and methods

### Analysis of *K*. *lactis* DK and homologous protein sequences

*K*. *lactis* DK protein homologs were determined using BLAST [26] and NCBI non-redundant protein sequence database (2020-09-19 version used). All homologous protein sequences with a similarity score E-value below 0.01 to DK were extracted from NCBI database, filtered to not greater than 70% sequence identity (nr70 sequence set of homologs obtained) using cd-hit software [27] and finally were aligned to *K*. *lactis* DK sequence according to the results obtained using BLAST (see S1 Table). The structures of the *K*. *lactis*, *S*. *cerevisiae*, *C*. *elegance* and human DK were predicted using AlphaFold 3 [24]. The 5 AlphaFold3 structural models of *K*. *lactis* DK were used for prediction of DK N- and C-terminal domain boundaries applying DomPred [28] and Merizo [29] programs. Amino acid sequence alignment of *K*. *lactis*, *S*. *cerevisiae*, *H*. *sapiens* and *C*. *elegans* DK presented in the S1 Fig was obtained from the alignment of the AlphaFold3 3D models of the DK proteins using USCF Chimera tools [30]. The representation of the alignment was carried out using ESPript3 program [31].

### Construction of plasmids

All DNA manipulations and cloning experiments followed standard protocols [32] or adhered to manufacturer’s recommendations. Enzymes and kits were purchased from UAB Thermo Fisher Scientific Baltics (Vilnius, Lithuania).

The *KlSEC59* and *S*. *cerevisiae SEC59* gene sequences were amplified by PCR using Phusion® High-Fidelity DNA Polymerase (Thermo Fisher Scientific, Baltics) and isolated chromosomal DNA of WGI and BY4741 strains as template. These sequences were then inserted into the pJET1.2 vector and used for cloning and construction of *KlSEC59* and *ScSEC59* expression cassettes. The corresponding forward and reverse oligonucleotide primers as well as primers for other DNA amplifications performed in this study are presented in the S2 Table. For construction of expression cassettes, each intermediate plasmid (pJET-KlSEC59 and pJET-ScSEC59) was amplified by PCR using Phusion® High-Fidelity DNA Polymerase (Thermo Fisher Scientific, Baltics) with corresponding forward and reverse primers (S2 Table), respectively. After ligation of the amplified DNA fragments into circular pJET-KlS59k and pJET-ScS59k plasmids, both expression cassettes, containing *K*. *lactis* or *S*. *cerevisiae SEC59* gene promoter and terminator sequences separated by two Aar I restriction endonuclease (RE) sites, allowing precise replacement of the yeast DK coding sequence (CDS) to the different DK CDSs, were obtained. The constructed *KlSEC59* and *ScSEC59* expression cassettes were sequenced and inserted into Sal I RE site of pADS and pRS426 vectors (Table 1).

**Table 1:** The list of plasmids constructed in this study.

Plasmid name	Description of the plasmid composition
**pKDP**	Previously described plasmid [21] which includes the *KlPGK1* gene promoter and terminator and the *KlURA3* gene
**pKDP-KlN**	Contains CDS of the *K*. *lactis* DK N-terminal domain (KlN) under *KlPGK1* promoter for expression in *K*. *lactis*
**pKDP-KlC**	Contains CDS of the *K*. *lactis* DK C-terminal domain (KlC) under *KlPGK1* promoter for expression in *K*. *lactis*
**pADS vector**	Constructed by insertion of *B*. *amyloliquefaciens* α-amylase gene from the previously described pBori-AMY plasmid [33] into the pKDU7 vector which contains the *KlURA3* gene [34].
**pJET-KlS59k**	Contains *KlSEC59* expression cassette where *K*. *lactis KlSEC59* gene promoter and terminator sequences are separated by two Aar I RE sites, allowing precise replacement of the yeast DK coding sequence (CDS) to the different CDSs, in pJET1.2 vector.
**pJET-ScS59k**	Contains *ScSEC59* expression cassette where *S*. *cerevisiae SEC59* gene promoter and terminator sequences are separated by two AarI RE sites, allowing precise replacement of the yeast DK CDS to the different CDSs, in pJET1.2 vector.
**pADKS vector**	Constructed by insertion of the *KlSEC59* expression cassette from pJET-KlS59k plasmid into the pADS vector Sal I RE site; used for insertion and expression of different DK and its domains CDSs under *KlSEC59* promoter for expression in *K*. *lactis*
**pADS-KlDK**	Contains the original *KlSEC59* (KlDK) gene
**pADS-KlDKwss**	Contains the *KlSEC59* WSS mutant allele (KlDKwss)
**pADS-ScDK**	Contains the original Sc*SEC59* (ScDK) gene
**pADKS-KlN**	Contains CDS of the *K*. *lactis* DK N-terminal domain (KlN) under *KlSEC59* promoter for expression in *K*. *lactis*
**pADKS-KlC**	Contains CDS of the *K*. *lactis* DK C-terminal domain (KlC) under *KlSEC59* promoter for expression in *K*. *lactis*
**pADKS-KlN-KlC**	Contains CDSs of the *K*. *lactis* DK N- and C-terminal domains (KlN and KlC) for individual co-expression under *KlSEC59* promoters
**pADKS-CeN/KlC**	Contains CDS of the chimeric DK protein CeN/KlC constructed after fusion of CDSs of the *C*. *elegans* DK N- (CeN) and the *K*. *lactis* DK C-terminal (KlC) domains under *KlSEC59* promoter for expression in *K*. *lactis*
**pADKS-KlN/CeC**	Contains CDS of the chimeric DK protein KlN/CeC constructed after fusion of CDS of the *K*. *lactis* DK N- (KlN) and the *C*. *elegans* DK C-terminal (CeC) domains under *KlSEC59* promoter for expression in *K*. *lactis*
**pADKS-ScDK**	Contains CDS of the *S*. *cerevisiae* DK (ScDK) under *KlSEC59* promoter for expression in *K*. *lactis*
**pADKS-CeDK**	Contains CDS of the *C*. *elegans* DK (CeDK) under *KlSEC59* promoter for expression in *K*. *lactis*
**pADKS-HsDK**	Contains CDS of the *H*. *sapiens* DK (HsDK) under *KlSEC59* promoter for expression in *K*. *lactis*
**pRS426-KlDK**	Contains the original *K*. *lactis* (KlDK) *KlSEC59* gene
**pRS426-KS vector**	Constructed by insertion of the *KlSEC59* expression cassette pJET-KlS59k plasmid into the pRS426 vector Sal I RE site for expression in *S*. *cerevisiae*
**pRS426-KS-KlN**	Contains CDS of the *K*. *lactis* DK N-terminal domain (KlN) under *KlSEC59* promoter for expression in *S*. *cerevisiae*
**pRS426-KS-KlC**	Contains CDS of the *K*. *lactis* DK C-terminal domain (KlC) under *KlSEC59* promoter for expression in *S*. *cerevisiae*
**pRS426-KS-KlN-KlC**	Contains CDSs of the *K*. *lactis* DK N- and C-terminal domains (KlN-KlC) for individual co-expression under *KlSEC59* promoters
**pRS426-SS**	Constructed by insertion of the Sc*SEC59* expression cassette pJET-ScS59k plasmid into the pRS426 vector Sal I RE site; used for insertion and expression of different DK CDSs in *S*. *cerevisiae*
**pRS426-SS-KlDK**	Contains CDS of the *K*. *lactis* DK (KlDK) under Sc*SEC59* promoter for expression in *S*. *cerevisiae*
**pRS426-SS-CeDK**	Contains CDS of the *C*. *elegans* DK (CeDK) under Sc*SEC59* promoter for expression in *S*. *cerevisiae*
**pRS426-SS-HsDK**	Contains CDS of the *H*. *sapiens* DK (HsDK) under Sc*SEC59* promoter for expression in *S*. *cerevisiae*
**pKgR1**	Constructed after deletion of *K*. *lactis* Cas9 expressing cassette from the previously described pKCR1 plasmid [21] providing resistance to geneticin.
**pKgR-LK1/2**	Contains two guide RNA (gRNA) expressing cassettes [21] with specific linkers K1 and K2 (S2 Table) for direction of Cas9 to the 5’ and 3’ ends of the *K*. *lactis KlSEC59* CDS used for DK gene replacement in *K*. *lactis* chromosome
**pKgR-LS1/2**	Contains two gRNA expressing cassettes [21] with linkers S1 and S2 (S2 Table) for direction of Cas9 to the 5’ and 3’ ends of the *S*. *cerevisiae ScSEC59* CDS used for *S*. *cerevisiae* DK CDS replacement in *K*. *lactis*
**pKgR-LS1/2-KS-KlN**	Contains two gRNA expressing cassettes with linkers S1 and S2 and the *K*. *lactis* DK N-terminal domain (KlN) expressing cassette
**pKPD-C9**	Constructed after insertion of Cas9 CDS under *KlPGK1* gene promoter into the previously described pKDP plasmid [21] which includes a *KlURA3* gene
**pFG1**	Constructed after deletion of the *GAL10-PYK1* expression cassette from the previously described pFGG3 vector providing resistance to formaldehyde [35]
**pFGgR2-LS1/2**	Contains two gRNA expressing cassettes with specific linkers S1 and S2 (S2 Table) for direction of Cas9 to the 5’ and 3’ ends of the *S*. *cerevisiae ScSEC59* CDS for DK CDS replacement in *S*. *cerevisiae*

The CDSs of human (HsDK sequence ID: NP_055723.1) and *C*. *elegans* (CeDK sequence ID: NP_001355372.1) DK were codon-optimized for expression in yeast *S*. *cerevisiae* and *de novo* synthesized commercially by BioCat GmbH (Heidelberg Germany). Some deviations from strict, high-frequency codon usage were introduced to facilitate the insertion or removal of RE recognition sites.

The CDSs of *K*. *lactis* and *S*. *cerevisiae* DK and CDSs of *K*. *lactis* DK N- (1–281 aa) and C- (282–515 aa) terminal domains were amplified by PCR. All amplified CDSs had introduced Aar I RE recognition sites for cloning and ATG and TAA codons when needed. The chimeric DK protein KlN/CeC CDS was constructed after fusion of PCR amplified N-terminal domain (1–295 aa) CDS of the *K*. *lactis* DK with PCR amplified CDS of C- terminal domain (77–299 aa) of the *C*. *elegans* DK. The second chimeric DK protein CeN/KlC CDS was constructed after fusion of PCR amplified CDS of the *C*. *elegans* DK N-terminal domain (1–76 aa) with PCR amplified CDS of the *K*. *lactis* DK C-terminal domain (296–515 aa). All amplification reactions were performed using Phusion® High-Fidelity DNA Polymerase (Thermo Fisher Scientific, Baltics) and all amplified CDSs after cloning were sequenced.

All different DK encoding CDSs were placed under *KlSEC59* and/or *ScSEC59* promoters in the *KlSEC59* and/or *ScSEC59* expression cassettes in the pADKS or pRS427 vectors. All plasmids constructed in this study are listed in Table 1.

### Strains, media, and growth conditions

All recombinant plasmids were constructed in the *Escherichia coli* strain DH5α. Bacterial cells were cultured in LB broth supplemented with 50 μg/mL of ampicillin.

The yeast strains used in this study are as follows: *K*. *lactis* strain CBS2359 (MAT*a*) derivatives, WGI (MATa Δ*ura3*) strain and WSS (MATa Δ*ura3 sec59*) strain with DK mutations G405S and I419S, described previously [21] sourced from our laboratory collection, *S*. *cerevisiae* BY4741 (MAT*a his3Δ1 leu2Δ0 met15Δ0 ura3Δ0*)) strain, obtained from Thermo Fisher Scientific.

Yeast cells were cultured in YPD medium (1% yeast extract, 2% soy peptone and 2% glucose) at 30°C or other temperatures as indicated. *S*. *cerevisiae* transformants with pFGgR2-LS1/2 plasmid (Table 1) were plated and then selected on YPD medium with 2% agar, supplemented with 0.3 μL/mL or 0.6 μL/mL formaldehyde (37%) respectively and transformants with pADS and pKPD-C9 plasmids (Table 1) were selected on synthetic YNB medium (0.67% yeast nitrogen base, 2% glucose, 1% casamine acids/5% amino acid solution (0.04% adenine sulphate, 0.04% L-tryptophan, 0.04% L-histidine HCl, 0.04% L-arginine HCl, 0.04% L-methionine, 0.06% L-tyrosine, 0.06% L-leucine, 0.06% L-lysine HCl, 0.1% L-phenylalanine, 0.2% L-glutamic acid, 0.2% L-aspartic acid, 0.3% L-valine, 0.4% L-threonine, 0.8% L-serine) and 2% agar). *K*. *lactis* URA+ transformants were selected on YNB medium (2% glucose, 0.67% yeast nitrogen base and 1% casamine acids). Yeast transformants with pKgR-LK1/2 and pKgR-LS1/2 plasmids (Table 1) were selected on YPD media supplemented with 100 μg/mL G418. The resistance to drugs was tested growing yeast cells on YPD or YNB medium supplemented with CFW 20 μg/mL or tunicamycin 0.3 μg/mL at 24°C, 30°C and 34°C or 30°C and 37°C for 2–4 days.

### Construction of yeast strains using CRISPR-Cas9 technology

The replacement of DK coding sequences in chromosomes of yeast *K*. *lactis* strain WGI and *S*. *cerevisiae* BY4741 strain was performed using CRISPR-Cas9 technology in two steps Firstly, *K*. *lactis* WGI strain was transformed with pKgR-LK1/2 plasmid containing two guide RNA (gRNA) expressing cassettes from pKCR1 and pKCR2 plasmids [21] with inserted specific linkers K1 and K2 (S2 Table) for direction of Cas9 to the 5’ and 3’ ends of the *K*. *lactis KlSEC59* CDS (Table 1). Thereafter, the selected geneticin resistant transformants were co-transformed with pKPD-C9 plasmid expressing *Streptococcus pyogenes* Cas9 endonuclease gene and marker-free donor DNA fragments PCR amplified from pADKS-ScDK, pADKS-HsDK, pADKS-CeDK and pADKS-KlC plasmids (Table 1). Transformants were selected on YNB medium supplemented with 100 μg/mL G418. The PCR-amplified replaced DK encoding CDS fragments of few selected transformants were sequenced.

For construction of the *K*. *lactis* strains in which CDS of *KlSEC59* gene was replaced with the CDS of KlC, and chimeric DK CeN/KlC or KlN/CeC, we utilized newly constructed Kl-ScDK strain transformed with the pKgR-LS1/2-KS-KlN or pKgR-LS1/2 plasmids containing two gRNA expressing cassettes [21] with inserted specific linkers S1 and S2 (S2 Table) for direction of Cas9 to the 5’ and 3’ ends of the *S*. *cerevisiae ScSEC59* CDS (Table 1). The geneticin-resistant transformants were co-transformed with the pKPD-C9 and marker-free donor DNA fragments were PCR amplified from pADKS-KlC, pADKS-KlN/CeC or pADKS-CeN/KlC plasmids (Table 2). The PCR-amplified fragments of the replaced DK CDS from selected transformants grown on YNB medium supplemented with 100 μg/mL G418 were sequenced.

**Table 2:** The list of newly engineered *K*. *lactis* and *S*. *cerevisiae* strains using CRISPR-Cas9 technology.

Yeast strain name	Description of the introduced changes
***K*. *lactis***	
**KlC-N**	The CDS of chromosomal gene *KlSEC59* in *K*. *lactis* was replaced with the CDS of C-terminal domain of *K*. *lactis* DK and expressed together with the *K*. *lactis* N-terminal domain CDS carried on the plasmid pKgR-LS1/2-KS-KlN.
**KlC-N-C**	The CDS of chromosomal gene *KlSEC59* in *K*. *lactis* was replaced with the CDS of C-terminal domain of the *K*. *lactis* DK and expressed alongside with the N-terminal domain CDS carried on the plasmid pKgR-LS1/2-KS-KlN and additional copies of the *K*. *lactis* C-terminal domain CDS carried on the plasmid pADKS-KlC.
**KlC-C**	The CDS of chromosomal gene *KlSEC59* in *K*. *lactis* was substituted with the CDS of C-terminal domain of the *K*. *lactis* DK and was expressed alongside with additional copies of the *K*. *lactis* C-terminal domain CDS, carried on the plasmid pADKS-KlC.
**Kl-ScDK**	The CDS of chromosomal gene *KlSEC59* in *K*. *lactis* was substituted with the CDS of *S*. *cerevisiae* DK.
**Kl-HsDK**	The CDS of chromosomal gene *KlSEC59* in *K*. *lactis* was substituted with the CDS of human DK.
**Kl-CeDK**	The chromosomal gene *KlSEC59* in *K*. *lactis* was substituted with the CDS of the nematode *C*. *elegans* DK.
**Kl-KlN/CeC**	The CDS of chromosomal gene *KlSEC59* in *K*. *lactis* was replaced with the CDS of the chimeric DK KlN/CeC.
**Kl-CeN/KlC**	The CDS of chromosomal gene *KlSEC59* in *K*. *lactis* was replaced with the CDS of the chimeric DK CeN/KlC.
***S*. *cerevisiae***	
**WSGS**	The chromosomal gene *SEC59* in *S*. *cerevisiae* was modified by introduction of the G407S and L421S DK mutations encoding changes. The *S*. *cerevisiae* DK mutations G407S and L421S match the DK mutations G405S and I419S of the *K*. *lactis* WSS strain (refer to S1 File and S1 Fig).
**Sc-KlDK**	The CDS of chromosomal gene *SEC59* in *S*. *cerevisiae* was replaced with the CDS of *K*. *lactis* DK.
**Sc-HsDK**	The CDS of chromosomal gene *SEC59* in *S*. *cerevisiae* was replaced with the CDS of human DK.
**Sc-CeDK**	The CDS of chromosomal gene *SEC59* in *S*. *cerevisiae* was replaced with the CDS of nematode *C*. *elegans* DK.

To replace DK coding sequences in the chromosomes of the *S*. *cerevisiae* BY4741 strain, yeast cells were transformed with pFGgR2-LS1/2 plasmid with two gRNA expressing cassettes [21] with inserted specific linkers S1 and S2 (S2 Table) for direction of Cas9 to the 5’ and 3’ ends of the S. *cerevisiae ScSEC59* CDS (Table 1). Thereafter, the selected formaldehyde-resistant transformants were co-transformed with mix of pKPD-C9 plasmid and marker-free donor DNA fragments PCR amplified from pRS426-SS-KlDK, pRS426-SS-CeDK, pRS426-SS-HsDK, plasmids (Table 1). Transformants were selected on YNB medium. The replaced DK encoding gene fragments of few selected transformants were PCR-amplified and sequenced. All constructed *K*. *lactis* and *S*. *cerevisiae* strains are listed in Table 2.

### Detection of CPY glycoforms

Exponentially growing *K*. *lactis* and *S*. *cerevisiae* cells were collected by centrifugation, and 50 mg of cells were suspended in 150 μL Y-PER Yeast Protein Extraction Reagent (Thermo Fisher Scientific, Rockford, IL, USA) containing 0.2 M phenylmethanesulfonyl fluoride (PMSF; BioChemica, AppliChem, Darmstadt, Germany). The suspended cells were disrupted by vortexing at room temperature for 20 min, followed by centrifugation and mixing of the collected supernatant with 2X SDS-PAGE electrophoresis sample buffer (1M Tris pH 6.8, 50% glycerol, 10% SDS, 0.5% bromophenol blue, 10% β-mercaptoethanol) followed by boiling for 10 min. The prepared samples were utilized for 7% SDS-PAGE electrophoresis and Western blot analysis, as previously described [36]. Primary polyclonal rabbit anti-CPY (carboxypeptidase C; PRC1) antibodies (GeneTex, Irvine, CA, USA) were diluted to 1:600, and secondary HRP-labeled anti-rabbit IgG conjugate antibodies (Bio-Rad, Hercules, CA, USA) were diluted to 1:3500. The Western blots were developed using 3,3′,5,5′-Tetramethylbenzidine (TMB) Liquid Substrate System for Membranes (Sigma-Aldrich, Saint Louis, MO, USA).

## Results

### Comparison of DK homologous protein sequences and structural model predicted by AlphaFold3 suggests two-domain structure of *K*. *lactis* DK

**Fig 1:**
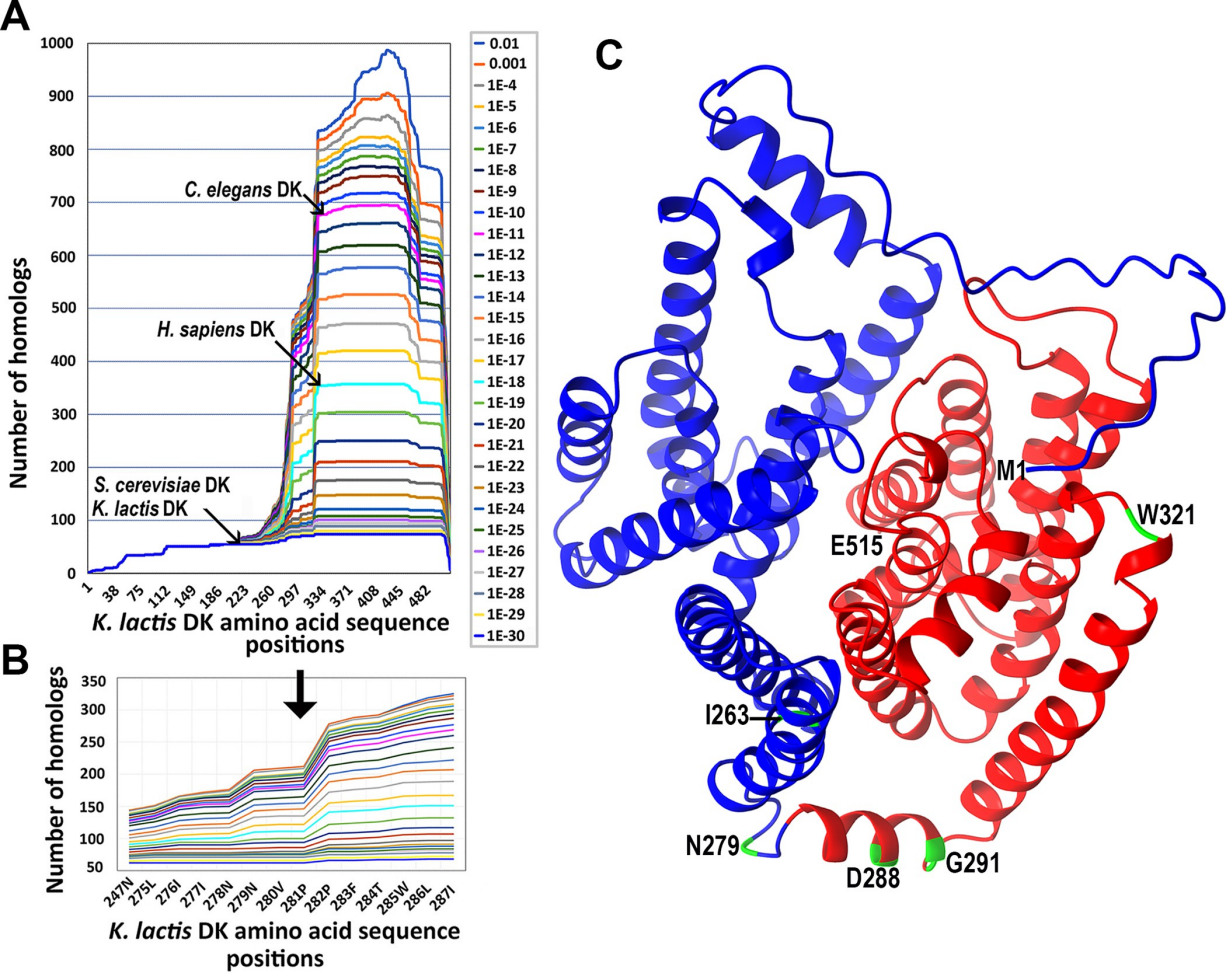
Prediction of *K*. *lactis* DK domain boundaries. (A, B) Analysis of the *K*. *lactis* DK sequence coverage by homologous regions from homologous proteins across varying sequence similarity stringencies. (A) Arrows indicate the positions of the yeasts *K*. *lactis* and *S*. *cerevisiae*, along with *H*. *sapiens* and *C*. *elegans* DK, based on their E-values; (B) The magnified coverage curve profile highlights the N/C-terminal domain separation site of the *K*. *lactis* DK at the domain boundary region; (C) Structural model of *K*. *lactis* DK predicted by AlphaFold3. The proposed N-terminal (blue, aa 1–281) and C-terminal (red, aa 282–515) domains are suggested based on evolutionary data. Potential domain boundary positions are highlighted in green at amino acid positions 263, 291, 288, and 279, as predicted using the Merizo approach for five AlphaFold3 structural models ([pTM = 0.88, average PAE = 0.76]). An additional domain boundary at position 321 was predicted with DomPred.

The search for DK homologs has revealed their widespread presence across all three living kingdoms. Therefore, homologous protein sequences were extracted from the NCBI database with a similarity score E-value below 0.01 to *K*. *lactis* DK. Following pairwise comparisons of the extracted sequences with the *K*. *lactis* DK sequence using the BLAST program, the sequences were sorted into bins based on E-value thresholds and categorized by levels of similarity. The coverage of the *K*. *lactis* DK sequence, based on homologous regions identified at various stringencies of sequence similarity is presented in Fig 1A. The results of this analysis, which examined DK sequence evolution across wide variety of organisms, suggest that *K*. *lactis* DK most likely comprises two distinct structural/functional domains. The C-terminal domain, responsible for the dolichol kinase function, shows homology to proteins across broad range of eukaryotes, archaea, and even bacteria (for more details, refer to S1 Table). In contrast, the N-terminal domain appears to be species-specific and may play a role in regulatory or other unknown auxiliary functions. The profile of the coverage curve (see Fig 1A, and its magnification in Fig 1B) indicates that the most plausible splitting point between the two domains lies between prolines 281 and 282 in the *K*. *lactis* DK sequence. This conclusion is supported by the sequence region starting at residue P282, which demonstrates a marked increase in homologs compared to other possible division points near the transition zone between the low-coverage (N-terminal part) and high-coverage (C-terminal part) regions.

To support our preliminary prediction that DK consists of two domains, we applied the DomPred method [28] and one of the most accurate domain boundary prediction tools to date, Merizo [29]. We tested the later on AlphaFold3 structural models of the *K*. *lactis* DK. Both methods predicted the presence of two structural domains in the *K*. *lactis* DK. DomPred predicted the domain boundary at amino acid position 321, while the Merizo approach, applied to five AlphaFold3 structural models, predicted boundaries at positions of 263, 291, 291, 288, and 279 amino acids (Fig 1C). Given that the region near amino acid position 320 (aa 331–346) in the *S*. *cerevisiae* DK was previously identified as responsible for the substrate (dolichol) binding [37], and considering our identification of the suppression mutation E319K, which restored the effects of the G407S and L421S DK mutations in the WSGS strain (see S1 File), we chose not to split domains at the 320 aa boundary position predicted by DomPred. Instead, we selected position at 281 aa, as suggested by the analysis of homologous DK protein sequences. This choice aligns with the domain boundaries predicted by the Merizo approach (Fig 1C). Interestingly, despite the species-specific nature of the non-homologous N-terminal domains, these domains are preserved in DK proteins across eukaryotes, archaea, and bacteria, with exception of nematodes, including *C*. *elegans* (S1 Fig). In nematodes, the DK protein is shorter and lacks an obvious N-terminal domain or possesses a very short one (1–76 aa).

### The DK C-terminal domain maintains the protein glycosylation level necessary for the survival of *K*. *lactis* only under overexpression

**Fig 2:**
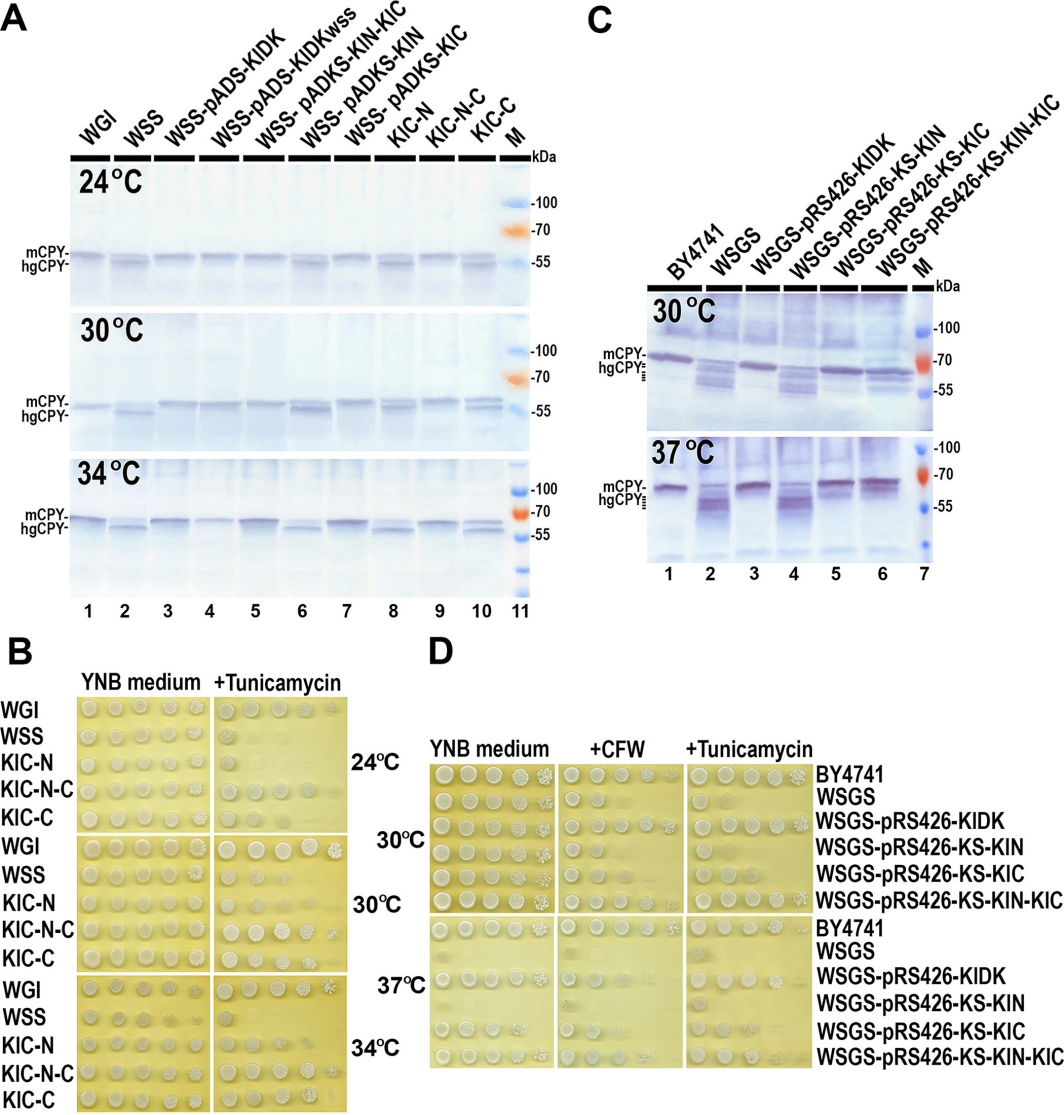
Phenotypic characterization of *K*. *lactis* and *S*. *cerevisiae* strains overexpressing additional copies of *K*. *lactis* DK or its domain-encoding sequences at different temperatures. Results from one out of four independent experiments are presented. **(A)** Western blot analysis showing the glycosylation status of CPY in cell lysates of *K*. *lactis* DK WSS and KIC strains overexpressing additional copies of different alleles of *K*. *lactis* DK or its domain CDS. The positions of the mature form of CPY (mCPY) and of the hypoglycosylated form/s (hgCPY) are indicated. M: Protein weight marker (Thermo Fisher Scientific Baltics). **(B)** Cell growth analysis of *K*. *lactis* KIC strain overexpressing additional copies of *K*. *lactis* DK N and C terminal domain CDS in response to drugs and temperature. **(C)** Western blot analysis showing the glycosylation status of CPY in cell lysates of *S*. *cerevisiae* WSGS mutant expressing additional copies of *K*. *lactis* DK N and C-terminal domain CDS. **(D)** Cell growth analysis of the *S*. *cerevisiae* of WSDS mutant expressing additional copies of *K*. *lactis* DK N- and C-terminal domain CDS in response to drugs and temperature.

Following the results of DK domains boundary prediction, we separated plausible N- (1–281 aa) and C- terminal (282–515 aa) domains of *K*. *lactis* DK by placing their encoding DNA fragments under the *KlPGK*1 and the *KlSEC59* promoters in the pKDP and the pADKS plasmids (Table 1). We examined their ability to restore CPY glycosylation in the *K*. *lactis* WSS strain by Western blotting. Reduced CPY glycosylation in *K*. *lactis* WSS mutant due to the DK G405S and I419S aa mutations has been demonstrated previously [21]. The hypoglycosylation manifested as the emergence of glycoforms lacking one or two N-glycan chains [21]. Thus, we utilized CPY glycosylation profiles as a metric to assess the impact of DK variants on the cellular glycosylation levels. However, yeast cells of WGI strain as well as WSS mutant transformed with pKDP-KlC plasmid harboring C terminal domain under strong constitutive *KlPGK1* promoter survived only for a few days after transformation suggesting that excessive overexpression of C terminal domain was toxic to the yeast cells. The overexpression of N terminal domain was not toxic under both promoters, *KlPGK1* and *KlSEC59*, but it was not able to restore CPY glycosylation in WSS mutant (Fig 2A, lane 6). On the other hand, the mild and controlled overexpression of the C terminal domain under the *KlSEC59* promoter was not toxic in both strains transformed with pADKS-KlC plasmid and restored CPY glycosylation in the WSS mutant at all three tested temperatures (Fig 2A, lane 7). A similar improvement in CPY glycosylation was observed in WSS strain transformed with the following plasmids: pADS-KlDK, harboring the intact *KlSEC59* gene; pADS-KlDKwss, harboring the mutant *Klsec59* gene; and pADKS-KlN-KlC, harboring separated C- and N-terminal domains under the *KlSEC59* promoter (Fig 2A, lanes 3–5). These results revealed that not only the DK C-terminal domain and intact DK but also an excess of mutated DK molecules could restore glycosylation of CPY to the WT level in the WSS mutant.

To eliminate the DK dose effect, we decided to replace the CDS of original *KlSEC59* gene in the *K*. *lactis* chromosome with a DK C-terminal domain-encoding fragment. Unfortunately, our initial attempts were unsuccessful. This outcome indicated that while the C-terminal domain was able to restore CPY glycosylation alongside mutated DK when overexpressed from the multicopy pADKS-KlC plasmid (Table 1), it could not perform the same function when expressed from a single chromosomal copy. This suggestion was partially confirmed after the successful replacement of the *KlSEC59* gene CDS with a C-terminal domain-encoding fragment in the chromosome of the *K*. *lactis* WGI strain, carrying the pKgR-LS1/2-KS-KlN plasmid (Table 1) expressing the N-terminal domain. Although DK activity in this newly constructed KlC-N yeast strain (Table 2) was relatively low, as evident by the presence of significant amounts of hypoglycosylated CPY (Fig 2A, line 8), it was sufficient to sustain yeast cell growth at all three tested temperatures. Moreover, the pKgR-LS1/2-KS-KlN plasmid expressing the N-terminal domain was very stable and could not be lost even over the course of several rounds of reinoculation. However, this plasmid could be replaced in the KlC-N strain with the pADKS-KlC plasmid (Table 1), ensuring the overexpression of the C-terminal domain. Interestingly, the CPY glycosylation level in this new KlC-C strain (Table 2) showed further improvement compared to the KlC-N strain but still did not reach the wild-type level (Fig 2A, lane 10). Nevertheless, in the intermediate yeast strain KlC-N-C (Table 2), where both N and C terminal domains were overexpressed, CPY glycosylation was restored to the WT level (Fig 2A, lane 9). As expected, the lower activity of DK resulted in slightly increased sensitivity of KIC-N and KIC-C strains, but not the KIC-N-C strain, to tunicamycin (Fig 2B). Tunicamycin is a mixture of homologous nucleoside antibiotics that inhibits certain enzymes, including UDP-N-acetyl-glucosamine-1-P (UDP-GlcNAc-P) transferase, which transfers GlcNAc-P from UDP-GlcNAc to Dol-P to form GlcNAc-PP-Dol in the ER [38]. These results confirmed that the activity of the DK C-terminal domain expressed from the single copy of its encoding fragment in the chromosome was not sufficient to maintain the protein glycosylation level necessary for survival of *K*. *lactis* cells. However, this deficiency was improved by additionally provided and overexpressed N- or C-terminal domain.

The ability of *K*. *lactis* DK N- and C-terminal domains to restore phosphorylation of dolichol was also tested in yeast *S*. *cerevisiae* WSGS strain with DK mutations G407S and L421S which match the DK mutations G405S and I419S of the *K*. *lactis* WSS strain (refer to S1A Fig and S1 File). The *S*. *cerevisiae* WSGS mutant was transformed with the plasmids pRS426-KS-KlN, pRS426-KS-KlC, pRS426-KS-KlN-KlC (Table 1), ensuring overexpression of the *K*. *lactis* DK and its N-, C-terminal domains, and co-expression of individual N- and C-terminal domains. The hypoglycosylation of *S*. *cerevisiae* CPY manifest as the emergence of glycoforms lacking one to four N-glycan chains. The analysis of CPY glycosylation changes in *S*. *cerevisiae* WSGS transformants revealed that the *K*. *lactis* DK C-terminal domain alone, as well as expressed together with the N-terminal domain, was able to restore CPY glycosylation and resistance to calcofluor-white (CFW) and tunicamycin in *S*. *cerevisiae* WSGS strain (Fig 2C and 2D). Nevertheless, the overexpression of the *K*. *lactis* DK C-terminal domain alone only partially improved the glycosylation of CPY, but the co-expression of both N- and C-terminal domains restored CPY glycosylation almost to the wild-type level at both tested temperatures (Fig 2C). This demonstrates that even separately expressed, both *K*. *lactis* DK N- and C-terminal domains were able to interact and function together, allowing the restoration of C-terminal domain activity to a level similar to intact DK activity in the WT strain.

### Human and nematode DKs, expressed from a single copy in the yeast chromosome, can complement DK functions in both *K*. *lactis* and *S*. *cerevisiae*

**Fig 3:**
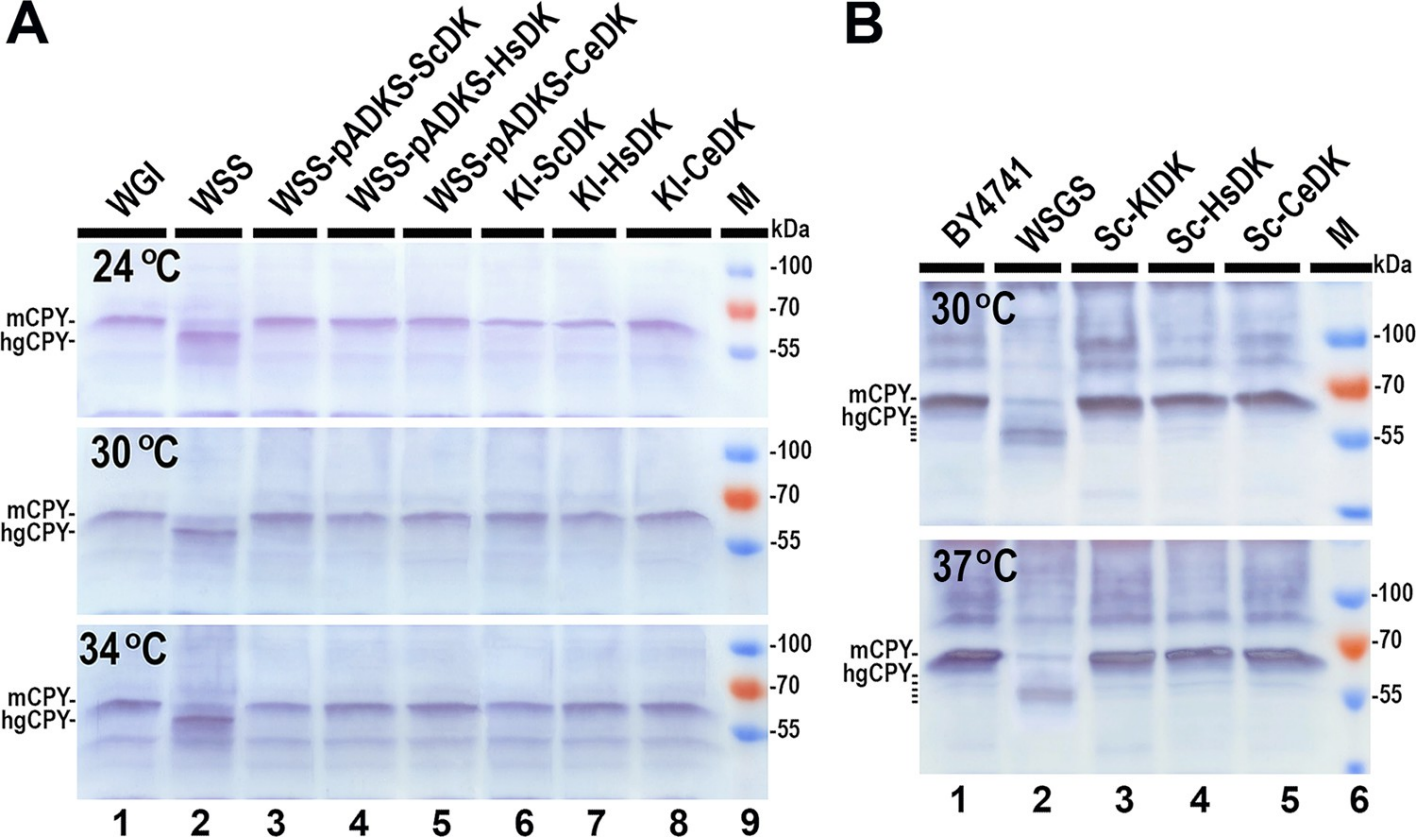
Western blot analysis showing the glycosylation status of CPY in cell lysates of *K*. *lactis* and *S*. *cerevisiae* strains expressing *S*. *cerevisiae*, *K*. *lactis*, human, or *C*. *elegans* DK. Results from one out of three independent experiments are presented. **(A)** Western blot analysis indicating the glycosylation status of CPY in *K*. *lactis* strains Kl-ScDK, Kl-HsDK, Kl-CeDK expressing *S*. *cerevisiae*, human, or *C*. *elegans* DK from the only copy of its encoding fragment in the chromosome and in the WSS mutant overexpressing these genes **(B).** Western blot analysis also indicates the glycosylation status of CPY in *S*. *cerevisiae* strains Sc-KlDK, Sc-HsDK, Sc-CeDK expressing *K*. *lactis*, human, or *C*. *elegans* DK from the only copy of it encoding fragment in the chromosome. The positions of the mature form of CPY (mCPY) and of the hypoglycosylated form/s (hgCPY) are indicated. M: Protein weight marker (Thermo Fisher Scientific Baltics).

Given that the ability of the *K*. *lactis* DK C-terminal domain or even the mutant *Klsec59* gene to maintain protein glycosylation at the required level was dose-dependent, it was intriguing to investigate if human or nematode *C*. *elegans* DK, with non-homologous N-terminal domains, expressed from a single gene copy in the yeast genome (with the yeast DK native gene removed) would be able to complement it (S1 Fig). First, we assessed the ability of *S*. *cerevisiae*, human, and nematode *C*. *elegans* DKs to restore CPY glycosylation in a *K*. *lactis* WSS mutant transformed with multicopy plasmids pADKS-ScDK, pADKS-HsDK, and pADKS-CeDK (Table 2). These plasmids harbor synthetic CDS of both human and nematode DK proteins and *S*. *cerevisiae SEC59* gene under the *KlSEC59* promoter. Western blot results confirmed that all three overexpressed recombinant DK proteins restored CPY glycosylation in a WSS mutant similarly to the *K*. *lactis KlSEC59* gene expressed from the plasmid at all three tested temperatures (Fig 3A, lanes 3–5 and Fig 2A lane 3).

However, in this experiment, recombinant DKs synthesized in the WSS strain acted alongside the original *K*. *lactis*, albeit mutated, DK with the native N-terminal domain. To ensure that the cloned recombinant DKs alone can complement the native yeast DK, we employed CRISPR-Cas9 technology to replace the *K*. *lactis* and *S*. *cerevisiae* DK encoding sequences in the chromosomes of both yeasts with the CDSs of human, nematode *C*. *elegans*, and *S*. *cerevisiae*, or *K*. *lactis* DKs under the *KlSEC59* or *SEC59* promoter, respectively. The analysis of CPY glycosylation levels in the constructed Kl-ScDK, Kl-HsDK, Kl-CeDK, Sc-KlDK, Sc-HsDK, and Sc-CeDK yeast strains (Table 2 and Fig 3) revealed that all recombinant DKs, expressed from a single copy in the yeast genome with the native yeast DK gene removed, fully complemented the DK functions in both yeast species at all three tested temperatures. This was observed regardless of whether their N- terminal domains were homologous (both yeast species DK), non-homologous (human and nematode DK), or very short (nematode DK).

### The N- and C-terminal domains of DK from different origins fused together form active chimeric DK

**Fig 4:**
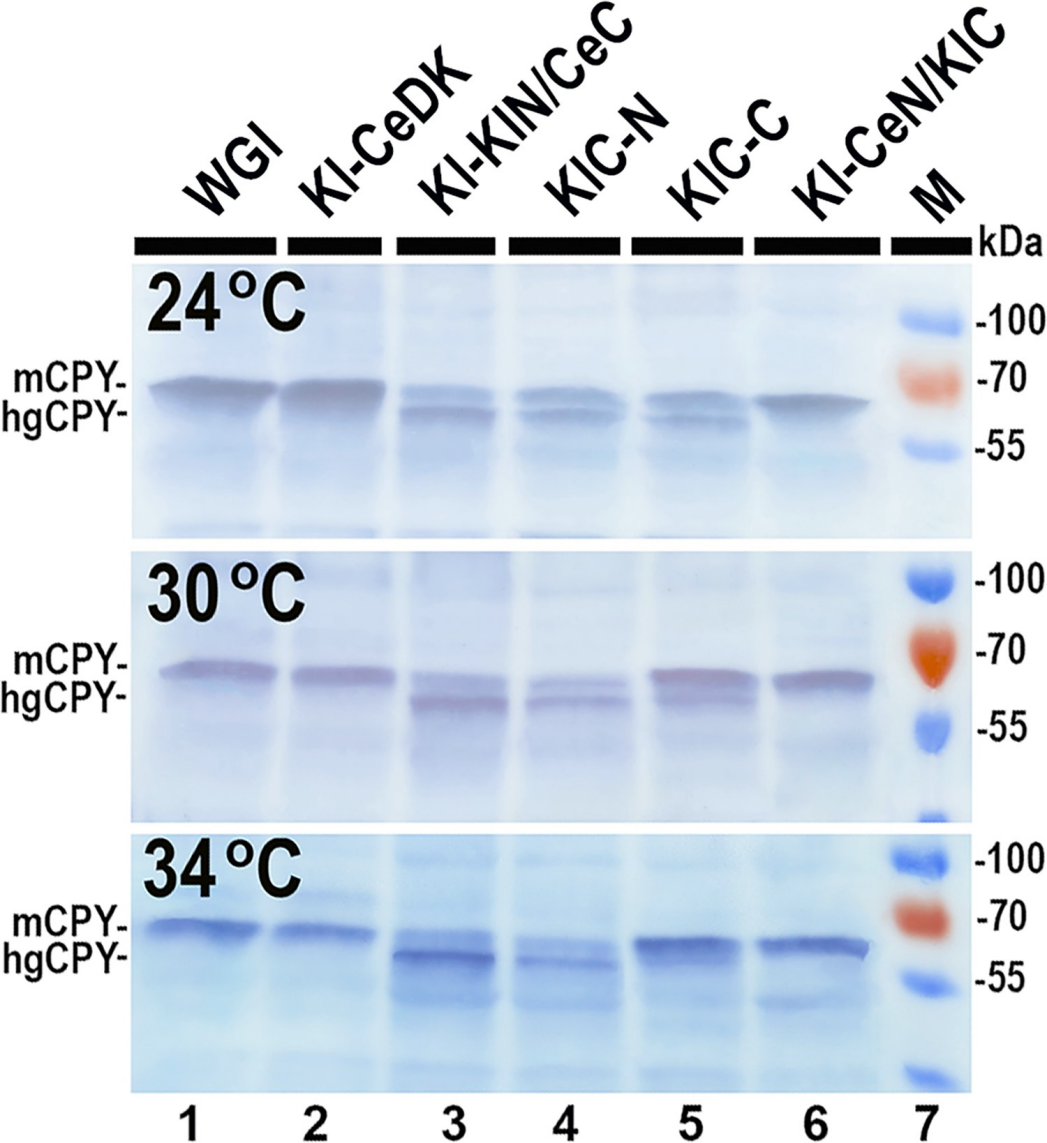
Western blot analysis showing the glycosylation status of CPY in cell lysates of *K*. *lactis* strains expressing chimeric DKs at different temperatures. The positions of the mature form of CPY (mCPY) and of the hypoglycosylated form (hgCPY) are indicated. M: Protein weight marker (Thermo Fisher Scientific Baltics). Results from one out of four independent experiments are presented.

To support our hypothesis regarding the two domains in the DK structure, we created two chimeric DK versions. In one, the N-terminal domain of *K*. *lactis* DK was fused with the C-terminal domain of nematode DK, while in the other, the N-terminal domain of nematode DK was fused with the C-terminal domain of *K*. *lactis* DK. Both chimeric DK genes, KlN-CeC and CeN-KlC, expressed from the multicopy plasmids, successfully restored CPY glycosylation in the *K*. *lactis* WSS mutant at all three tested temperatures. Subsequently, we employed CRISPR-Cas9 technology to replace the native CDS of *KlSEC59* gene on *K*. *lactis* chromosome with the CDS of chimeric DK, either KlN/CeC or CeN/KlC. The engineered *K*. *lactis* strains, Kl-CeN/KlC and Kl-KlN/CeC (Table 2), each carried a single copy of the chimeric DK gene in the chromosome, proving DK activity sufficient to support yeast cell growth. While both chimeric KlN/CeC and CeN/KlC genes successfully complemented yeast DK functions, the DK activity in Kl-KlN/CeC yeast strain was lower compared to the Kl-CeN/KlC strain or Kl-CeDK strain with the native DK allele from nematodes as the Kl-KIN/CeC strain contained significant amounts of hypoglycosylated CPY (Fig 4, lane3). On the contrary, the attachment of the short native nematode N-domain to the *K*. *lactis* DK C-terminal domain notably enhanced its activity. While the C-terminal domain alone could keep protein glycosylation sufficient to sustain yeast cell growth only upon overexpression, the chimeric CeN/KlC DK, expressed from a single copy in the yeast chromosome, maintained CPY glycosylation levels close to wild type at all tested temperatures (Fig 4, lanes 5, 6).

## Discussion

N-linked protein glycosylation is a prevalent protein modification observed across all three domains of life [39,40]. In each system, the oligosaccharide assembly initiates with the attachment of nucleotide-activated monosaccharides to a phosphorylated isoprenoid lipid (bactoprenol in the bacterial systems and dolichol in archaea and eukaryotes). Through the phosphorylation of dolichol, the DK facilitates the transfer of monosaccharides to Dol-P, initiating the biosynthesis of dolichol-linked oligosaccharides [11]. This process occurs at the periplasmic membrane in prokaryotes or at the cytoplasmic side of the ER membrane in eukaryotes [40,41]. The both yeast species *S*. *cerevisiae* and *K*. *lactis* DKs are encoded by essential genes, therefore yeast cells can only tolerate a limited reduction in Dol-P levels as shown in conditional yeast DK mutants [16,21]. Studies of the conditional *S*. *cerevisiae sec59-1* mutant revealed a decrease in membrane Dol-P levels, reaching 48% of WT levels at the permissive temperature and dropping to below 10% at the restrictive temperature [16]. This suggests that Dol-P levels in membrane of the *S*. *cerevisiae* WSGS mutant might be similarly low or even lower as CPY glycosylation was more severely affected in the WSGS mutant than in the *sec59-1* mutant (refer to S1 File). The *K*. *lactis* WSS mutant carries analogous DK mutations as the *S*. *cerevisiae* WSGS mutant. Therefore, it is likely that the reduction in Dol-P levels in the WSS mutant also approaches around 50%. The evaluation of protein glycosylation changes in the *K*. *lactis* DK WSS mutant demonstrated that the reduced DK activity selectively affected protein glycosylation and yeast cells exhibited some tolerance to Dol-P deficiency through the adjustments in protein glycosylation (S2 File). However, this tolerance is limited under typical growth conditions, as the Dol-P deficiency in *K*. *lactis*, as well as in *S*. *cerevisiae* DK-mutant strains, becomes less tolerable or even intolerable when another enzyme involved in N-linked glycosylation is inhibited by tunicamycin (Fig 2B and 2D). Tunicamycin inhibits activity of UDP-GlcNAc-P transferase, which catalyzes the first step of lipid-linked oligosaccharides the synthesis on Dol-P [38]. Notably, the tolerance to Dol-P deficiency differs between multicellular and unicellular eukaryotes. In multicellular organisms, changes in protein glycosylation, particularly in aberrantly glycosylated secretory glycoproteins, can disturb crucial functions, such as intercellular interactions, receptor recognition, and signal transduction—processes vital for the development, differentiation, and physiology of a particular cell, tissue, or organism [42]. This glycan content heterogeneity has been linked to various diseases. For instance, impairment in protein glycosylation due to DK mutations often leads to neurological symptoms and nervous system defects commonly associated with congenital disorders of glycosylation [43,44]. Furthermore, some variations in tolerance to Dol-P deficiency were observed even among different yeast species. Specifically, the *S*. *cerevisiae* WSGS and *sec59-1* strains, which have lower DK activity, display sensitivity to CFW, whereas the corresponding *K*. *lactis* WSS mutant does not exhibit such sensitivity [20,21, S1 File]. This drug sensitivity may be linked to differences in the cell wall composition of the two yeast species, which are affected differently by changes in protein glycosylation. Additionally, we observed that CPY glycosylation in *K*. *lactis* DK mutants was sensitive to low temperatures [21], a sensitivity not observed in *S*. *cerevisiae* (S1 File).

The analysis of DK sequences from various eukaryotic, archaeal, and bacterial species revealed that the homologies among these protein sequences are predominantly found in the C-terminal regions of DK protein (Fig 1A). Despite an overall 43% identity between *K*. *lactis* and *S*. *cerevisiae* DK sequences, the highest similarity was observed in the C-terminal region where the catalytic DK domain resides (refer to S1 Fig). This similarity among the DK C-terminal domain sequences is also accompanied by structural and functional conservation of the DK C-terminal domain across eukaryotes (refer to S1 Fig). Previous and recent findings confirmed that the C-terminal domain is crucial for the ability of DK to phosphorylate dolichol and is essential for DK activity. Most of identified mutations in the C-terminal domain of both yeast and human DK typically impair its ability to phosphorylate dolichol [20,22,45,46]. While the overexpression of the DK C-terminal domain alone was sufficient to maintain dolichol phosphorylation levels necessary for yeast cells survival, the activity of incomplete DK that consisted solely of the C-terminal domain, only provided the minimal level of protein glycosylation required but could be improved by provided the N terminal domain (Fig 2A, lanes 9 and 10). This underscores the importance of the N-terminal domain for full reconstitution of the DK activity as demonstrated by co-expression the separated N- and C-terminal domains. The co-expressed individual N- and C-terminal domains were able to combine and form an active protein, fully restoring CPY glycosylation to wild-type levels in both *K*. *lactis*, and *S*. *cerevisiae* (Fig 2). In contrast to the high conservation of the C-terminal domain sequences, the N-terminal domain sequences of DK exhibited significant variability and were species-specific (Fig 1A). However, AlphaFold 3 predicted that the N-terminal domain structures of *K*. *lactis*, *S*. *cerevisiae*, and human DKs, despite their non-homologous amino acid sequences, are similar (refer to S1 Fig). Additionally, the structure of the significantly shorter nematode DK N-terminal domain also showed some similarities (refer to S1 Fig). This observation may explain why human or nematode DK, despite having non-homologous N-terminal sequences could fully complement DK functions in both tested yeast species (Fig 3).

Our results suggest that if N-terminal domain is necessary solely for DK stability or spatial regulation, the *C*. *elegans* DK N-terminal domain represents the minimal structure sufficient for DK protein stability and activity. This was demonstrated with the chimeric CeN/KlC DK, whose activity in the constructed yeast strain Kl-KlN/CeC was nearly identical to that of native *K*. *lactis* DK, as assessed by CPY glycosylation efficacy (Fig 4, lanes 1 and 6). Conversely, the chimeric DK KlN-CeC, consisting of fused the *K*. *lactis* N-terminal domain and the nematode DK C-terminal domain and expressed from the single chromosomal copy CDS, displayed activity similar to the DK in the KIC-N strain according CPY glycosylation efficacy (Fig 4 lanes 3 and 4). Thus, substitution of the nematode N-terminal domain with the longer *K*. *lactis* N-terminal domain resulted in decreased activity of the chimeric KlN/CeC DK compared to the native nematode DK (Fig 4 lanes 2 and 3). It is plausible that the restoration of full DK activity using longer N-terminal domains might require a more precise adaptation of the N- and C-terminal domains used for the fusion. On the other hand, the N-terminal domain may serve not only as a structural component but also might be involved in the regulation of DK activity. The *K*. *lactis* N-terminal domain might possess down-regulating features that reduce the activity of the DK C-terminal domain, leading to the decreased KlN/CeC DK activity compared to the native nematode DK. In contrast, the short nematode N-terminal domain lacks these negative regulatory elements, allowing CeN/KlC DK activity to remain comparable to that of *K*. *lactis* DK. This suggestion is consistent with data showing the H34N and E164D mutations in the N-terminal domain of DK, identified in certain *K*. *lactis* strains, did not exhibit a detectable negative effect on protein glycosylation efficiency [21]. However, the human DK C99S mutation leads to a wide spectrum of phenotypic disorders and is lethal in human embryos, underscoring the significance of the DK N-terminal domain in the higher eukaryotes possible involved in DK activity regulation function [45].

Interestingly that all DK homologs derived from eukaryotes, archaea, and bacteria, that we collected from the NCBI database (S1 Table) possess species-specific and significantly longer N-terminal domains compared to those of various nematode species. These longer N-terminal domains have been preserved throughout evolution and likely besides DK stabilization function fulfill other, possibly regulatory, functions as well. Notably, the strong constitutive overexpression of the *K*. *lactis* C-terminal domain–rather than the intact DK protein–was toxic to yeast cells, particularly to WT cells. This observation suggests that the activity of DK may be regulated also at the protein level.

## Conclusions

The experimental data support the prediction that *K*. *lactis* DK is composed of N- and C-terminal domains, as suggested by bioinformatics analyses of DK homologous sequences and domain boundary predictions by DomPred and Merizo approaches based on of AlphaFold3 structural models of *K*. *lactis* DK. While the C-terminal domain performs the essential DK function crucial for its activity, the N-terminal domain may contribute to the regulation of DK activity and act as a structural domain. The C-terminal domain of *K*. *lactis* DK alone can sustain the necessary protein glycosylation level for the survival of *K*. *lactis* cells only when overexpressed. Despite nonhomologous sequences and differing lengths of the N-terminal domains, the DKs of humans and *C*. *elegans*, expressed from the single copy of its encoding fragment in the chromosome, complement DK functions in both yeast species, *K*. *lactis* and *S*. *cerevisiae*. Furthermore, the N- and C-domains of *K*. *lactis* and *C*. *elegans* DK can interchange and form active chimeric DKs.

## Supporting information

S1 Fig Analysis of *K*. *lactis*, *S*. *cerevisiae*, *H*. *sapiens* and *C*. *elegans* DK sequence and structure similarities. (PDF)

S1 Table Analyzed DK homologues. (XLSX)

S2 Table Sequences of oligonucleotide primers used in this study. (PDF)

S1 File Analysis of protein glycosylation and secretion changes in the dolichol kinase mutants of *Saccharomyces cerevisiae*. (PDF)

S2 File Analysis of protein glycosylation profile in the *K*. *lactis* WSS mutant using a two-dimensional gel electrophoresis. (PDF)

S1 Raw images (TIF)

S2 Raw images (TIF)

## References

[1] Spiro RG. Protein glycosylation: nature, distribution, enzymatic formation, and disease implications of glycopeptide bonds. Glycobiology. 2002;12: 43R–56R. doi: 10.1093/glycob/12.4.43r 12042244

[2] Helenius A, Aebi M. Roles of N-linked glycans in the endoplasmic reticulum. Annu Rev Biochem. 2004;73: 1019–49. doi: 10.1146/annurev.biochem.73.011303.073752 15189166

[3] Lehle L, Strahl S, Tanner W. Protein glycosylation, conserved from yeast to man: a model organism helps elucidate congenital human diseases. Angew Chem Int Ed Engl. 2006;45: 6802–18. doi: 10.1002/anie.200601645 17024709

[4] Ohtsubo K, Marth JD. Glycosylation in cellular mechanisms of health and disease. Cell. 2006;126: 855–67. doi: 10.1016/j.cell.2006.08.019 16959566

[5] Denecke J, Kranz C. Hypoglycosylation due to dolichol metabolism defects. Biochim Biophys Acta. 2009; 1792(9):888–95. doi: 10.1016/j.bbadis.2009.01.013 19419701

[6] Aebi M. N-linked protein glycosylation in the ER. Biochim Biophys Acta. 2013;1833: 2430–2437. doi: 10.1016/j.bbamcr.2013.04.001 23583305

[7] Breitling J, Aebi M. N-linked protein glycosylation in the endoplasmic reticulum. Cold Spring Harb Perspect Biol. 2013;5:a013359. doi: 10.1101/cshperspect.a013359 23751184 PMC3721281

[8] Lommel M, Strahl S. Protein O-mannosylation: conserved from bacteria to humans. Glycobiology. 2009;19: 816–828. doi: 10.1093/glycob/cwp066 19429925

[9] Orlean P, Menon AK. Thematic review series: lipid posttranslational modifications. GPI anchoring of protein in yeast and mammalian cells, or: how we learned to stop worrying and love glycophospholipids. J Lipid Res. 2007;48: 993–1011. doi: 10.1194/jlr.R700002-JLR200 17361015

[10] Schenk B, Fernandez F, Waechter CJ. The ins(ide) and out(side) of dolichyl phosphate biosynthesis and recycling in the endoplasmic reticulum. Glycobiology. 2001;11: 61R–70R. doi: 10.1093/glycob/11.5.61r 11425794

[11] Fernandez F, Shridas P, Jiang S, Aebi M, Waechter CJ. Expression and characterization of a human cDNA that complements the temperature-sensitive defect in dolichol kinase activity in the yeast sec59-1 mutant: the enzymatic phosphorylation of dolichol and diacylglycerol are catalyzed by separate CTP-mediated kinase activities in *Saccharomyces cerevisiae*. Glycobiology. 2002;12: 555–62. 12213788 10.1093/glycob/cwf068

[12] Buczkowska A, Swiezewska E, Lefeber DJ. Genetic Defects in Dolichol Metabolism. J Inherit Metab Dis. 2015;38: 57–69. doi: 10.1007/s10545-014-9760-1 25270028 PMC4281381

[13] Kanehara K, Cho Y, Lin YC, Chen CE, Yu CY, Nakamura Y. Arabidopsis DOK1 encodes a functional dolichol kinase involved in reproduction. Plant J. 2015;81: 292–303. doi: 10.1111/tpj.12727 25406445

[14] Lindner H, Kessler SA, Müller LM, Shimosato-Asano H, Boisson-Dernier A, Grossniklaus U. TURAN and EVAN mediate pollen tube reception in *Arabidopsis synergids* through protein glycosylation. PLoS Biology. 2015;13:e1002139. 25919390 10.1371/journal.pbio.1002139 PMC4412406

[15] Meserve JH, Nelson JC, Marsden KC, Hsu J, Echeverry FA, Jain RA, et al. A forward genetic screen identifies Dolk as a regulator of startle magnitude through the potassium channel subunit Kv1.1. PLoS Genet. 2021;17:e1008943. doi: 10.1371/journal.pgen.1008943 34061829 PMC8195410

[16] Heller L, Orlean P, Adair WLJr. *Saccharomyces cerevisiae sec59* cells are deficient in dolichol kinase activity. Proc Natl Acad Sci USA. 1992;89: 7013–16. 1323123 10.1073/pnas.89.15.7013 PMC49635

[17] Ferro-Novick S, Novick P, Field C, Schekman R. Yeast secretory mutants that block the formation of active cell surface enzymes. J Cell Biol. 1984;98: 35–43. doi: 10.1083/jcb.98.1.35 6368571 PMC2113008

[18] Orlean P. Dolichol phosphate mannose synthase is required in vivo for glycosyl phosphatidylinositol membrane anchoring, O mannosylation, and N glycosylation of protein in *Saccharomyces cerevisiae*. Mol Cell Biol. 1990;10: 5796–805. 2146492 10.1128/mcb.10.11.5796-5805.1990 PMC361358

[19] Orlean P. Enzymes that recognize dolichols participate in three glycosylation pathways and are required for protein secretion. Biochem Cell Biol. 1992;70: 438–47. doi: 10.1139/o92-067 1333231

[20] Orłowski J, Machula K, Janik A, Zdebska E, Palamarczyk G. Dissecting the role of dolichol in cell wall assembly in the yeast mutants impaired in early glycosylation reactions. Yeast. 2007;24: 239–52. doi: 10.1002/yea.1479 17397129

[21] Ziogiene D, Valaviciute M, Norkiene M, Timinskas A, Gedvilaite A. Mutations of *Kluyveromyces lactis* dolichol kinase enhance secretion of recombinant proteins. FEMS Yeast Res. 2019;19(3), pii: foz024. 10.1093/femsyr/foz024 30865773

[22] Shridas P, Waechter CJ. Human Dolichol Kinase, a Polytopic Endoplasmic Reticulum Membrane Protein with a Cytoplasmically Oriented CTP-Binding Site. J Biol Chem 2006;281: 31696–704. doi: 10.1074/jbc.M604087200 16923818

[23] Haeuptle MA, Hennet T. Congenital Disorders of Glycosylation: An Update on Defects Affecting the Biosynthesis of Dolichol-Linked Oligosaccharides. Hum. Mutat. 2009;30: 1628–41. doi: 10.1002/humu.21126 19862844

[24] Abramson J, Adler J, Dunger J, Evans R, Green T, Pritzel A, et al. Accurate structure prediction of biomolecular interactions with AlphaFold 3. Nature. 2024 Jun;630(8016):493–500. doi: 10.1038/s41586-024-07487-w 38718835 PMC11168924

[25] Varadi M, Anyango S, Deshpande M, Nair S, Natassia C, Yordanova G, et al. AlphaFold Protein Structure Database: massively expanding the structural coverage of protein-sequence space with high-accuracy models. Nucleic Acids Res. 2022;50(D1): D439–D444. doi: 10.1093/nar/gkab1061 34791371 PMC8728224

[26] Altschul SF, Madden TL, Schäffer AA, Zhang J, Zhang Z, Miller W, et al. Gapped BLAST and PSI-BLAST: a new generation of protein database search programs. Nucleic Acids Res. 1997;25: 3389–402. doi: 10.1093/nar/25.17.3389 9254694 PMC146917

[27] Li W, Godzik A. Cd-hit: a fast program for clustering and comparing large sets of protein or nucleotide sequences. Bioinformatics. 2006;22: 1658–9. doi: 10.1093/bioinformatics/btl158 16731699

[28] Bryson K, Cozzetto D, Jones DT. Computer-assisted protein domain boundary prediction using the DomPred server. Curr Protein Pept Sci. 2007; 8(2):181–8. doi: 10.2174/138920307780363415 17430199

[29] Lau AM, Kandathil SM, Jones DT. Merizo: a rapid and accurate protein domain segmentation method using invariant point attention. Nat Commun. 2023; 19;14(1):8445. doi: 10.1038/s41467-023-43934-4 38114456 PMC10730818

[30] Pettersen EF, Goddard TD, Huang CC, Couch GS, Greenblatt DM, Meng EC, et al. UCSF Chimera—a visualization system for exploratory research and analysis. J Comput Chem. 2004; 25(13):1605–12. doi: 10.1002/jcc.20084 15264254

[31] Robert X, Gouet P. Deciphering key features in protein structures with the new ENDscript server. Nucleic Acids Res. 2014; 42(Web Server issue):W320–4. doi: 10.1093/nar/gku316 24753421 PMC4086106

[32] Sambrook J, Russell DW. Molecular cloning: A Laboratory Manual. Cold Spring Harbor, Cold Spring Harbor Press; 2001.

[33] Bartkeviciute D, Sasnauskas K. Studies of yeast *Kluyveromyces lactis* mutations conferring super-secretion of recombinant proteins. Yeast. 2003;20: 1–11. 12489121 10.1002/yea.935

[34] Bartkevičiūtė D, Šiekštelė R, Sasnauskas K. Heterologous expression of the *Kluyveromyces marxianus* endopolygalacturonase gene (EPG1) using versatile autonomously replicating vector for a wide range of host. Enzyme Microb Technol. 2000;26: 653–6. doi: 10.1016/s0141-0229(00)00155-1 10862869

[35] Slibinskas R, Samuel D, Gedvilaite A, Staniulis J, Sasnauskas K. Synthesis of the measles virus nucleoprotein in yeast *Pichia pastoris* and *Saccharomyces cerevisiae*. J Biotechnol. 2004;107: 115–24. 14711495 10.1016/j.jbiotec.2003.10.018

[36] Valaviciute M, Norkiene M, Goda K, Slibinskas R, Gedvilaite A. Survey of molecular chaperone requirement for the biosynthesis of hamster polyomavirus VP1 protein in *Saccharomyces cerevisiae*. Arch Virol. 2016;161: 1807–1819. 27038828 10.1007/s00705-016-2846-3

[37] Albright CF, Orlean P, Robbins PW. A 13-amino acid peptide in three yeast glycosyltransferases may be involved in dolichol recognition. Proc Natl Acad Sci USA. 1989;19: 7366–9. doi: 10.1073/pnas.86.19.7366 2678101 PMC298062

[38] Barnes G, Hanse WJ, Holcomb CL, Rine J. Asparagine-linked glycosylation in *Saccharomyces cerevisiae*: genetic analysis of an early step. Mol Cell Biol. 1984;4: 2381–8. 6096695 10.1128/mcb.4.11.2381-2388.1984 PMC369068

[39] Schwarz F, Aebi M. Mechanisms and principles of N-linked protein glycosylation. Curr Opin Struct Biol. 2011;21: 576–82. doi: 10.1016/j.sbi.2011.08.005 21978957

[40] Lechner J, Wieland F. Structure and biosynthesis of prokaryotic glycoproteins. Annu Rev Biochem. 1989;58: 173–94. doi: 10.1146/annurev.bi.58.070189.001133 2673008

[41] Swiezewska E, Danikiewicz W. Polyisoprenoids: structure, biosynthesis and function. Prog Lipid Res. 2005;44: 235–58. doi: 10.1016/j.plipres.2005.05.002 16019076

[42] Krištić J, Lauc G. Ubiquitous importance of protein glycosylation. Methods in Molecular Biology. Humana Press Inc; 2017. p. 1503. doi: 10.1007/978-1-4939-6493-2_1 27743354

[43] Scott H, Panin VM. The role of protein N-glycosylation in neural transmission. Glycobiology. 2014;24: 407–17. doi: 10.1093/glycob/cwu015 24643084 PMC3976283

[44] Freeze HH, Eklund EA, Ng BG, Patterson MC. Neurology of inherited glycosylation disorders. Lancet Neurol. 2012;11: 453–66. doi: 10.1016/S1474-4422(12)70040-6 22516080 PMC3625645

[45] Kranz C, Jungeblut C, Denecke J, Erlekotte A, Sohlbach C, Debus V, et al. A defect in dolichol phosphate biosynthesis causes a new inherited disorder with death in early infancy. Am J Hum Genet. 2007;80: 433–40. doi: 10.1086/512130 17273964 PMC1821118

[46] Lefeber DJ, de Brouwer APM, Morava E, Riemersma M, Schuurs-Hoeijmakers JHM, Absmanner B, et al. Autosomal recessive dilated cardiomyopathy due to DOLK mutations results from abnormal dystroglycan O-mannosylation. PLoS Genet. 2011;7(12):e1002427. doi: 10.1371/journal.pgen.1002427 22242004 PMC3248466

