# Supplementary material for: Dolichol kinases from yeast, nematode and human can replace each other and exchange their domains creating active chimeric enzymes in yeast
Source: PLoS One. 2024 Nov 7;19(11):e0313330. doi: 10.1371/journal.pone.0313330 (PMC11542857; doi:10.1371/journal.pone.0313330)
Supplement: S1 Fig — (PDF) [file pone.0313330.s001.pdf]

S1 Fig.

A

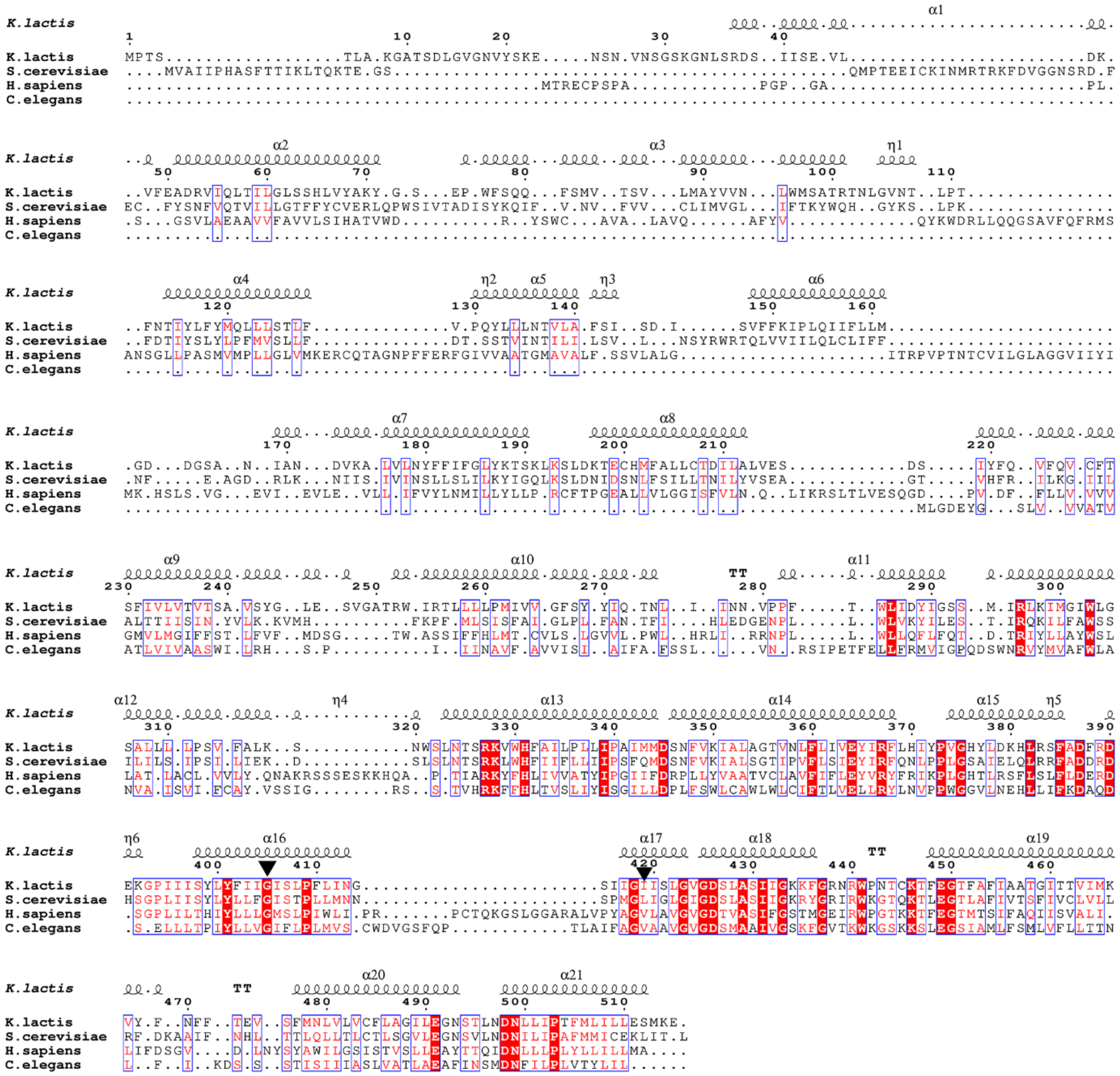
K.lactis MPTS..................TLA.KGATSDLGVGNVYSKE.....NSN.VNSGSKGNLSRDS..IISE.VL........................DK.
S.cerevisiae ....MVAIIPHASFTTIKLTQKTE.GS.......................................QMPTEEICKINMRTRKFDVGGNSRD.F
H.sapiens ......................................MTRECPSPA.............PGP..GA..........................PL.
C.elegans ....
K.lactis ..VFEADRVIQLTILGLSSHLVYAKY.G.S...EP.WFSQQ...FSMV..TSV...LMAYVVN...LWMSATRTNLGVNT..LPT...............
S.cerevisiae EC..FYSNFVQTVILLGTFFYCVERLQPWSIVTADISYKQIF..V.NV..FVV...CLIMVGL...IFTKYWQH..GYKS..LPK...............
H.sapiens .S...GSVLAEAAVVFAVVLSIHATVWD..........R..YSWC...AVA..LAVQ......AFYV.............QYKWDRLLQQGSAVFQFRMS
C.elegans ....
K.lactis ..FNTIYLFYMQLLLSTLF.............V.PQYLLLNTVLA.FSI..SD.I......SVFFKIPLQIIFLLM.......................
S.cerevisiae ..FDTIYSLYLPFMVSLLF.............DT.SSTVINTILI.LSV..L...NSYRWRTQLVVIILQLCLIFF.......................
H.sapiens ANSGLLPASMVMPLLGLVMKERCQTAGNPFFERFGIVVAATGMAVALF.SSVLALG....................ITRPVPTNTCVILGLAGGVIIYI
C.elegans ....
K.lactis .GD...DGSA..N..IAN...DVKA.LVLNYFFIFGLYKTSKLKSLDKTECHMFALLCTDILALVES............DS.....IYFQ..VFQV.CFT
S.cerevisiae .NF.....E.AGD..RLK....NIIS.IVINSLLSLILKYIGQLKSLDNIDSNLFSILLTNILYVSEA............GT.....VHFR..ILKG.IIL
H.sapiens MK.HSLS.VG...EVI..EVLE..VLL.IFVYLNMILLYLLP.RCFTPGEALLVLGGISFVLN.Q..LIKRSLTLVESQGD....PV.DF..FLLV.VVV
C.elegans ....MLGDEYG...SLV..VVATV
K.lactis SFIVLVTVTSA.VSYG..LE..SVGATRW.IRTLLLLPMIVV.GFSY.YIQ.TNL..I..INN.VPPF.....T..WLIDYIGSS..M.IRLKIMGIWLG
S.cerevisiae ALTTIISIN.YVLK.KVMH..........FKPF.MLSISFAI.GLPL.FAN.TFI....HLEDGENPL.....L..WLVKYILES..T.IRQKILFAWSS
H.sapiens GMVLMGIFFST.LFVF..MDSG.....TW.ASSIFFHLMT.CVLS.LGVVL.PWL..HRLI..RRNPL.....L..WLLQFLFQT..D.TRIYLLAYWSL
C.elegans ATLVIVAASWI.LRH...S.P..........I..IINAVF.AVVISI..AIFA.FSSL..L...VN.RSIPETFELLFRMVIGPQDSWNRVYMVAFWLA
K.lactis SALLL.LPSV.FALK..S...........NWSLNTSRKVWHFAILPLLIPAIMMDSNFVKIALAGTVNLFLIVEYIRFLHIYPVGHYLDKHLRSFADFRD
S.cerevisiae ILILS.IPSI.LIEK..D...........SLSLNTSRKLWHFIIFLLIIPSFQMDSNFVKIALSGTIPVFLSIEYIRFQNLPPLGSAIELQLRRFADDRD
H.sapiens LAT.LACL.VVLY.QNAKRSSSESKKHQA..P.TIARKYFHLIVVATYIPGIIFDRPLLYVAATVCLAVFIFLEYVRYFRIKPLGHTLRSFLSLFLDERD
C.elegans NVA.ISVI.FCAY.VSSIG........RS..S.TVHRKFFHLTVSLIYISGILLDPLFSWLCAWLWLCIFTLVELLRYLNVPPWGGVLNEHLLIFKDAQD
K.lactis EKGPIIISYLYFIIGISLPFLING.......................SIIGIISLGVGDSLASIIGKKFGRNRWPNTCKTFEGTFAFIAATGITTVIMK
S.cerevisiae HSGPLIISYLYLLFGISTPLLMNN.......................SPMGLIGLGIGDSLASIIGKRYGRIRWKGTQKTLEGTLAFIVTSFIVCLVLL
H.sapiens .SGPLILTHIYLLLGMSLPIWLI.PR.......PCTQKGSLGGARALVPYAGVLAVGVGDTVASIFGSTMGEIRWPGTKKTFEGTMTSIFAQIISVALI.
C.elegans .S.ELLLTPIYLLVGIFLPLMVS.CWDVGSFQP...........TLAIFAGVAAVGVGDSMAAIVGSKFGVTKWKGSKKSLEGSIAMLFSMLVFLLTTN
K.lactis VY.F..NFF..TEV..SFMNLVLVCFLAGILEGNSTLNDNLLIPTFMLILLESMKE.
S.cerevisiae RF.DKAAIF..NHL..TTLQLLTLCTLSGVLEGNSVLNDNILIPAFMMICEKLIT.L
H.sapiens LIFDSGV....D.LNYSYAWILGSISTVSLLEAYTTQIDNLLLPLYLLILLMA....
C.elegans L..F..I..KDS.S..STISIIIASLVATLAEAFINSMDNFILPLVTYLIL......

B

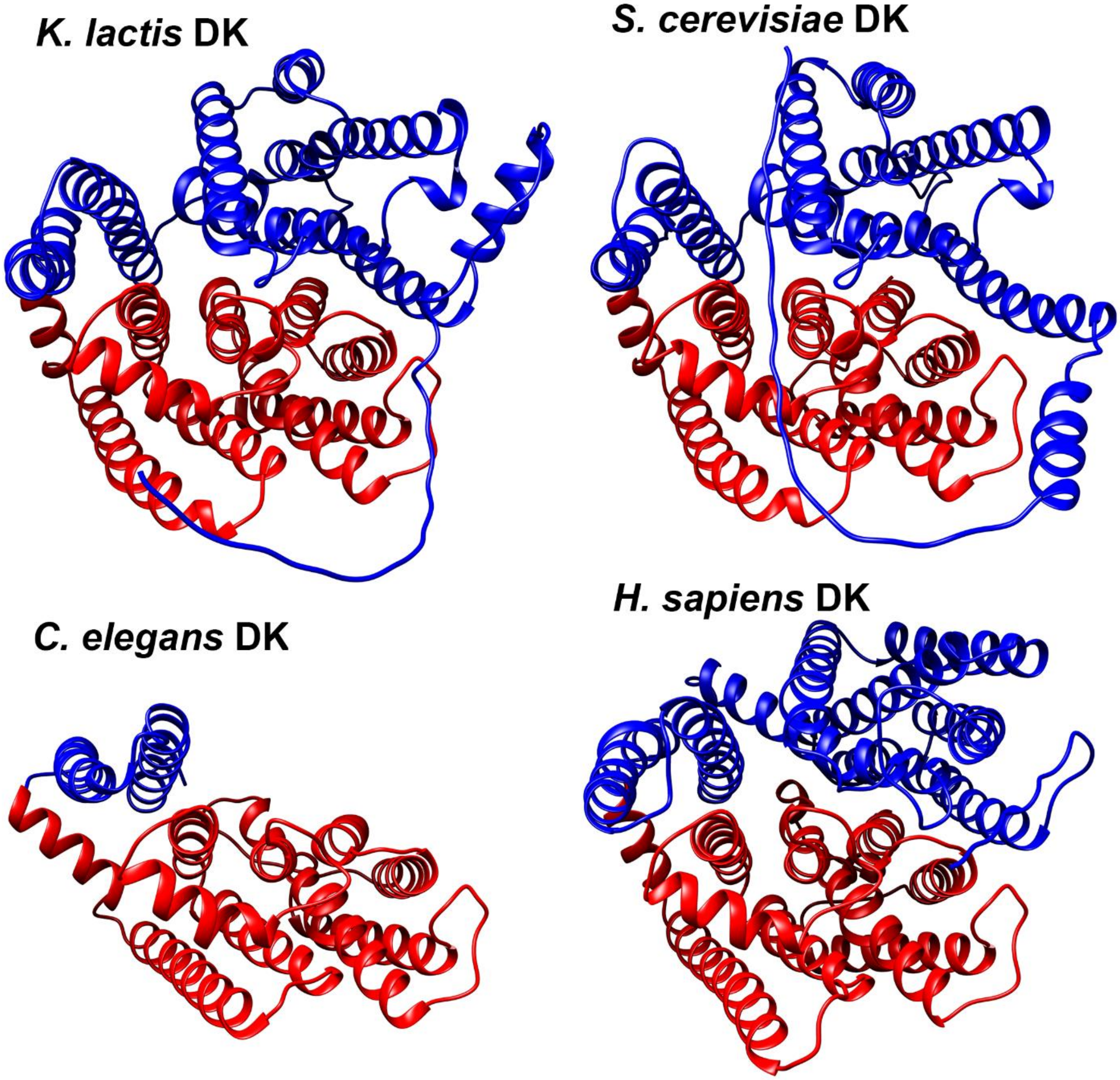

C

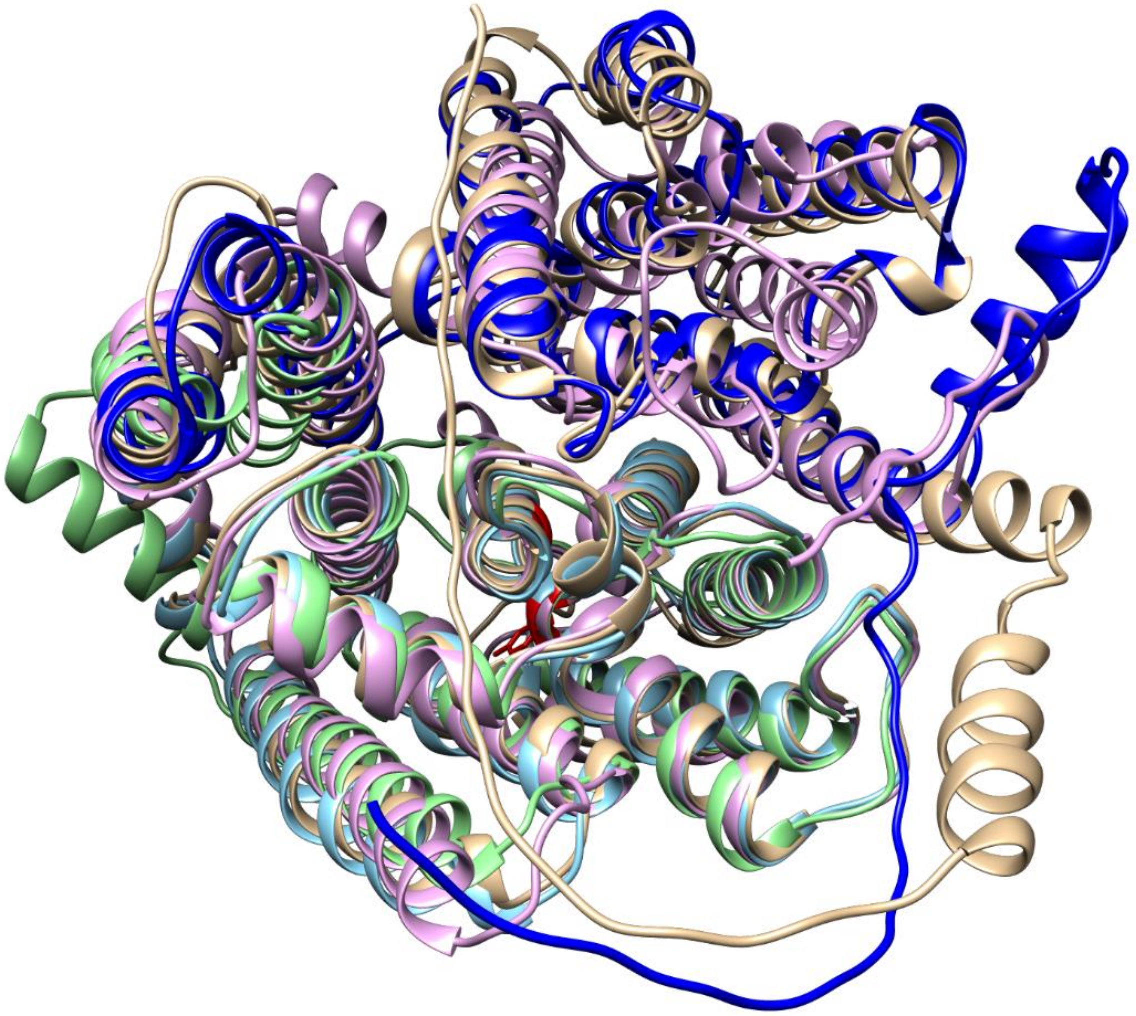

S1 Fig. Analysis of *K. lactis, S. cerevisiae, H. sapiens* and *C. elegans* DK sequence and structure similarities. (A) Amino acid sequence alignment of *K. lactis, S. cerevisiae, H. sapiens* and *C. elegans* DK obtained from the alignment of AlphaFold3 3D models of appropriate proteins using USCF Chimera tools. The aa residues G405, I419 which mutated in the *K.lactis* DK WSS mutant, and corresponding aa residues G407, L421 in *S.cerevisiae* WSGS mutant DK are marked with ▼. Representation of the alignment was carried out using ESPript3. (B) *K. lactis, S. cerevisiae, H. sapiens and C. elegans* DK 3D AF3 models obtained with AlphaFold3. The proposed N-terminal (blue) and C-terminal (red) domains are suggested based on evolutionary data. (C) Superimposed 3D structural models (obtained with AlphaFold3) of 4 dolichol kinases. *K.lactis* DK N-terminal – colored in blue and C-terminal domai -in sky blue, *S.cerevisiae* DK – in tan, *H.sapiens* – in plum, *C.elegans* – colored in light green. The aa residues G405, I419 which mutated in *K.lactis* DK WSS mutant, and aa residues G407, L421 which mutated in *S.cerevisiae* WSGS DK are marked in red.
