# Supplementary material for: Dolichol kinases from yeast, nematode and human can replace each other and exchange their domains creating active chimeric enzymes in yeast
Source: PLoS One. 2024 Nov 7;19(11):e0313330. doi: 10.1371/journal.pone.0313330 (PMC11542857; doi:10.1371/journal.pone.0313330)
Supplement: S2 Table — (PDF) [file pone.0313330.s003.pdf]

S2 Supporting Table. Sequences of oligonucleotide primers used in this study

Primer's name	Primer sequence (5'-3')	Application
**KD1**<br>**KR1**	CGAGCTCGTCGACGAAGTCATCACAAGTGATCAC<br>CGAGCTCGTCGACGATCATTGGTCCAGAATGAAC	Primers for amplification of the *KlSEC59* gene
**SD1**<br>**SR1**	CTCAGCCTTTCATAGCTCCCACTC<br>GCGATTCTTCCAACTCTAAGAGC	Primers for amplification of the *ScSEC59* gene
**KD2**<br>**KR2**	GCACCTGCTTGGCATATTAAGTTTGATTGC<br>GCACCTGCGGAATAATATTGATAGATAGTCAAACCTG	Primers for construction of *KlSEC59* expression cassette
**SD2**<br>**SR2**	GCACCTGCCGACCATATAGGCAATGAACAGAATAAC<br>GCACCTGCTCTTTAATCAAAGTCAATTATTTTCAAC	Primers for construction of *ScSEC59* expression cassette
**KD3**<br>**KR3**	GCACCTGCTTAATATGCCAACTTCCACGTTAGC<br>GCACCTGCCAATATTATTCCTTCATAGATTCCAACAATATC	Primers for amplification of the *KlSEC59* gene CDS for cloning into pJET-ScS59k
**SD3**<br>**SR3**	GCACCTGCCTAATATGGTCGCTATAATACCTC<br>GCACCTGCCAATATTAAAGAGTAATTAATTTTTCACAAATC	Primers for amplification of the *ScSEC59* gene CDS for cloning into pJET-KlS59k
**KD4**<br>**KR4**	GCACCTGCTTAATATGCCAACTTCCACGTTAGC<br>GCACCTGCCAATATTATGGGACATTGTTAATGATGAG	Primers for amplification of DK N domain encoding *KlSEC59* gene fragment for cloning into pJET-KlS59k
**KD5**<br>**KR5**	GCACCTGCTTAATATGTCTCCGTTCACATGGCTTATAG<br>GCACCTGCCAATATTATTCCTTCATAGATTCCAACAATATC	Primers for amplification of DK C domain encoding *KlSEC59* gene fragment for cloning into pJET-KlS59k
**KD6**<br>**KR6**<br>**CD1**<br>**CR1**	GCTCTAGAATGCCAACTTCCACGTTAGC<br>CACACCTGCTAAACTCTGATCATAGAGGAACCG<br>CGTCACCTGCGAATAGAGTTTATATGGTTGC<br>GCTCTAGATTACAAGATCAAATAAG	Primers for construction of hybrid KlN-CeC protein encoding DNA fragment
**KD3**<br>**CR3**	GCACCTGCTTAATATGCCAACTTCCACGTTAGC<br>CAGCACCTGCTAGAATTACAAGATCAAATAAGTAACCAATG	Primers for amplification of the hybrid KlN-CeC protein encoding CDS for cloning into pJET-KlS59k
**CD2**<br>**CR2**<br>**KD71**<br>**KR7**	CTGCACCTGCTTAATATGTTGGGTGATGAATATGGTTC<br>CTGCACCTGCACATGTCTATTCCAAGAATCTTG<br>CTGCACCTGCGATCAGACTGAAAATAATGG<br>GCACCTGCCAATATTATGGGACATTGTTAATGATGAG	Primers for construction of hybrid CeN-KlC protein encoding gene
**CD3**<br>**CR3**	CTGCACCTGCTTAATATGTTGGGTGATGAATATGGTTC<br>CAGCACCTGCTAGAATTACAAGATCAAATAAGTAACCAATG	Primers for amplification of the *CeSEC59* gene CDS for cloning into pJET-KlS59k and pJET-ScS59k
**KLA1**<br>**KLS1**	AATCAATGTAAACAGCGGATCTAA<br>AAACTTAGATCCGCTGTTTACATT	Linker K1 encoding 20 bp gRNA targeting *KlSEC59* sequence for replacement of *KlSEC59* CDS by CRISPR/Cas9
**KLA2**<br>**KLS2**	GATCGATATTGTTGGAATCTATGA<br>AAACTCATAGATTCCAACAATATC	Linker K2 encoding 20 bp gRNA targeting *KlSEC59* sequence for replacement of *KlSEC59* CDS by CRISPR/Cas9
**SLA1**<br>**SLS1**	AATCTATTCTGTTCATTGCCTATA<br>AAACTATAGGCAATGAACAGAATA	Linker S1 encoding 20 bp gRNA targeting *ScSEC59* sequence for replacement of *ScSEC59* CDS by CRISPR/Cas9
**SLA2**<br>**SLS2**	GATCTCACAAATCATCATAAATGC<br>AAACGCATTTATGATGATTTGTGA	Linker S2 encoding 20 bp gRNA targeting *ScSEC59* sequence for replacement of *ScSEC59* CDS by CRISPR/Cas9
**SD4**<br>**SR4**	CGTCACCTGCGGGATCATAGCGGGCCATTGATCATATC<br>GCACACCTGCCGCTATGATCCCTATCATCAGCAAACCTTC	Primers for introduction of DK PAM1 mutation into *ScSEC59* gene

**SD5**<br>**SR5**	CGTCACCTGCTCCCGAAAATTGGGTCATTTCATCATTTTC<br>GCACACCTGCCAATTTTCGGGAGGTGTTC	Primers for introduction of DK W332G mutation into *ScSEC59* gene
**SD6**<br>**SR6**	CGTCACCTGCATATCAACACCTTTATTAATG<br>GCACACCTGCAGGTGTTGATATTGAGAAGAGTAAATAAAG	Primers for introduction of DK G407S mutation into *ScSEC59* gene
**SD7**<br>**SR7**	CGTCACCTGCATAGGATTGGGAATTGGTG<br>GCACACCTGCTCCCAATCCTATTGAACCCATTGGAGAG	Primers for introduction of DK L421S mutation into *ScSEC59* gene
**SD8**<br>**SR8**	CGTCACCTGCGGATTGGGAATTGGTGATTCACTAGCATC<br>GCACACCTGCAATTCCCAATCCTATTAGATCCATTGGAGAG	Primers for introduction of DK G420D and PAM2 silent mutation into *ScSEC59* gene
**SLA3**<br>**SLS3**	GATCATATGATCAATGGCCCGCTG<br>AAACCAGCGGGCCATTGATCATAT	Linker S3 encoding 20 bp gRNA targeting *ScSEC59* sequence for its replacement into mutant alleles of *ScSEC59* gene by CRISPR/Cas9
**SLA4**<br>**SLS4**	GATCTTTACCAATAATAGATGCTA<br>AAACTAGCATCTATTATTGGTAAA	Linker S4 encoding 20 bp gRNA targeting *ScSEC59* sequence for its replacement into mutant allele (G420D) of *ScSEC59* gene by CRISPR/Cas9
**SD9**<br>**SR1**	GAAGGGCATTATCCTTGCTCTCAC<br>GCGATTCTTCCAACTCTAAGAGC	Primers for amplification of the *ScSEC59* gene donor DNA fragment
**SD1**<br>**SR9**	CTCAGCCTTTCATAGCTCCCACTC<br>GACGTTAAACCTGAATTGGCTAAC	Primers for amplification of the *ScSEC59* gene for sequencing
