# Supplementary material for: Dolichol kinases from yeast, nematode and human can replace each other and exchange their domains creating active chimeric enzymes in yeast
Source: PLoS One. 2024 Nov 7;19(11):e0313330. doi: 10.1371/journal.pone.0313330 (PMC11542857; doi:10.1371/journal.pone.0313330)
Supplement: S1 File — (PDF) [file pone.0313330.s004.pdf]

S1 File

Analysis of protein glycosylation and secretion changes in the dolichol kinase mutants of *Saccharomyces cerevisiae*

Introduction

After identification of the DK G407S and L421S mutations in the *KlSEC59* gene of the *K. lactis* MD2/1-9 strain [1], we opted to generate *S. cerevisiae* BY 4741 DK mutant strains carrying the corresponding mutations in the *SEC59* gene (Table S1-1; S1 Fig. A). Additionally, we constructed *S. cerevisiae* BY 4741 mutants with several other *sec59* mutations (Table S1-1). The *S. cerevisiae sec59-1* conditional mutant was isolated as a secretion mutant characterized by considerably reduced N-linked glycosylation and secretion of CPY, invertase, and alpha-factor [2]. Interestingly, the different authors identified distinct mutations of the same *sec59-1* allele, either W332G [3] or G420D [4], prompting us to generated yeast mutants with both alleles. We examined the impact of *S. cerevisiae* DK mutations on CPY glycosylation, α-amylase secretion as well as yeast cell growth rate and sensitivity to drugs at different temperatures in all constructed *S. cerevisiae sec59* mutants.

Table S1-1. The list of generated *S. cerevisiae* BY4741 strain derivatives harboring the respective *SEC59* mutations.

S. cerevisiae strain	Short strain name	*SEC59* mutations	DK amino acid substitution	Published yeast strains with corresponding DK mutations
BY4741-GGGL	**GGGL**	TGG to GGT	W332G	*S. cerevisiae sec59-1* [3]
BY4741-WSGL	**WSGL**	GGA to TCA	G407S	*K. lactis* MD2/1-9 [1]
BY4741-WGDL	**WGDL**	GGT to GAT	G420D	*S. cerevisiae sec59-1* [4]
BY4741-WGGS	**WGGS**	CTA to TCA	L421S	*K. lactis* MD2/1-9 [1]
BY4741-WSGS	**WSGS**	GGA to TCA and CTA to TCA	G407S and L421S	*K. lactis* MD2/1-9 [1]
BY4741-K	**BY-K**	GAG to AAG	E319K	This study
BY4741-WSGS-K	**WSGS-K**	GAG to AAG	E319K	This study

Results and discussion

Construction of *sec59* mutant strains.

Initially, the plasmid pJET-ScSEC59 was amplified by PCR using Phusion® High-Fidelity DNA Polymerase (Thermo Fisher Scientific, Baltics, Vilnius, Lithuania) for introduction of silent PAM site 1 mutation into *SEC59* gene encoding *S. cerevisiae* DK employing corresponding primers (S2 Table). Subsequently, this altered plasmid pJET-ScSEC59-1 was further amplified by PCR utilizing the corresponding primers encoding DK mutations W332G, G407S, L421S (S2 Table). Additionally, the plasmid pJET-ScSEC59-407S, featuring the introduced G407S mutation, was amplified with corresponding primers to introduce the L421S mutation, facilitating the generation of a *sec59* mutant allele with both G407S and L421S mutations (S2 Table). The DK mutation G420D was introduced together with PAM site 2 mutation by PCR amplification of pJET-ScSEC59 plasmid using the respective primers (S2 Table). The plasmids constructed in this manner, bearing different *sec59* mutant alleles (pJET-ScSEC59-332G, pJET-ScSEC59-407S, pJET-ScSEC59-420D, pJET-ScSEC59-421S, and pJET-ScSEC59-SS), were employed for the amplification of marker-free donor DNA fragments. For replacement of WT *SEC59* gene to mutant alleles (see Table S1-1) in the chromosomes of the *S. cerevisiae* BY4741 strain a plasmid pFGgR1 was constructed. This involved modifying the pFGG3 vector, which includes a segment of the yeast 2μm plasmid and the *FDH1* gene of *Candida maltosa*, providing resistance to formaldehyde [5]. The GAL10-PYK1 expression cassette in pFGG3 vector was substituted with a chimeric gRNA expression cassette featuring *S. cerevisiae* SNR52 RNA [1]. Following the insertion of specific *SEC59* 20 bp target site sequence encoding linkers, S3 and S4, the plasmids pFGgR1-LS3 and pFGgR1-LS4 were constructed. The replacement of the *SEC59* gene with mutant alleles in the chromosome of the yeast strain BY4741 was performed in two sequential steps. Firstly *S. cerevisiae* cells were transformed with pFGgR1-LS3 or pFGgR1-LS4 plasmid. Thereafter, the selected transformants displaying resistance to

formaldehyde were co-transformed with a mixture of the pKPD-C9 plasmid expressing the *S. pyogenes* Cas9 endonuclease gene (Table 1) and marker-free donor DNA fragments. These DNA fragments were PCR amplified from the pJET-ScSEC59-332G, pJET-ScSEC59-407S, pJET-ScSEC59-420D, pJET-ScSEC59-421S, and pJET-ScSEC59-SS plasmids using SD9 and SR1 primers (S2 Table). Transformants were selected on YNB medium with formaldehyde. Subsequently, the PCR-amplified gene fragments encoding DK from a subset of selected transformants were sequenced. The *S. cerevisiae* strains, featuring with replaced mutant *sec59* alleles, were identified and are listed in Table S1-1.

Phenotypic characterization of generated *sec59* mutant strains

We investigated the impact of each DK mutation (W332G, G407S, G420D, L421S, and both G407S and L421S) on glycosylation of CPY and the α-amylase secretion in all five constructed strains (Table S1-1) This analysis aimed to enhance our understanding of how these mutations affected the activity of the DK protein. Additionally, we assessed the sensitivity of mutant yeast cells to various drugs, providing insights into potential changes in cell wall composition resulting from defects in N-linked glycosylation, O-linked glycosylation, and GPI anchor synthesis.

The CPY glycosylation defects were clearly evident in the WGDL and WSGS strains and marginally detectable in GGGL strain at all four tested temperatures, with only subtle emergence in the WSGL and WGGS strains (Fig S1-1A). Additionally, the WGDL and WSGS strains displayed sensitivity to the higher growth temperatures, 34°C or 37°C (Fig S1-1B). As anticipated, the observed glycosylation defects correlated with the sensitivity of mutant strains to tunicamycin (Fig S1-1B). This sensitivity was temperature-dependent, with the least impact on the growth of mutant yeast cells at 24°C. Specifically, the WSGS strain exhibited high sensitivity to tunicamycin across all tested temperatures, while the WGDL and GGGL strains showed higher sensitivity at 34°C or 37°C but reduced sensitivity at 24°C or 30°C. The WSGL strain

displayed sensitivity to tunicamycin only at 34°C and 37°C, while the WGGS strain exhibited sensitivity only at 37°C (Fig S1-1B).

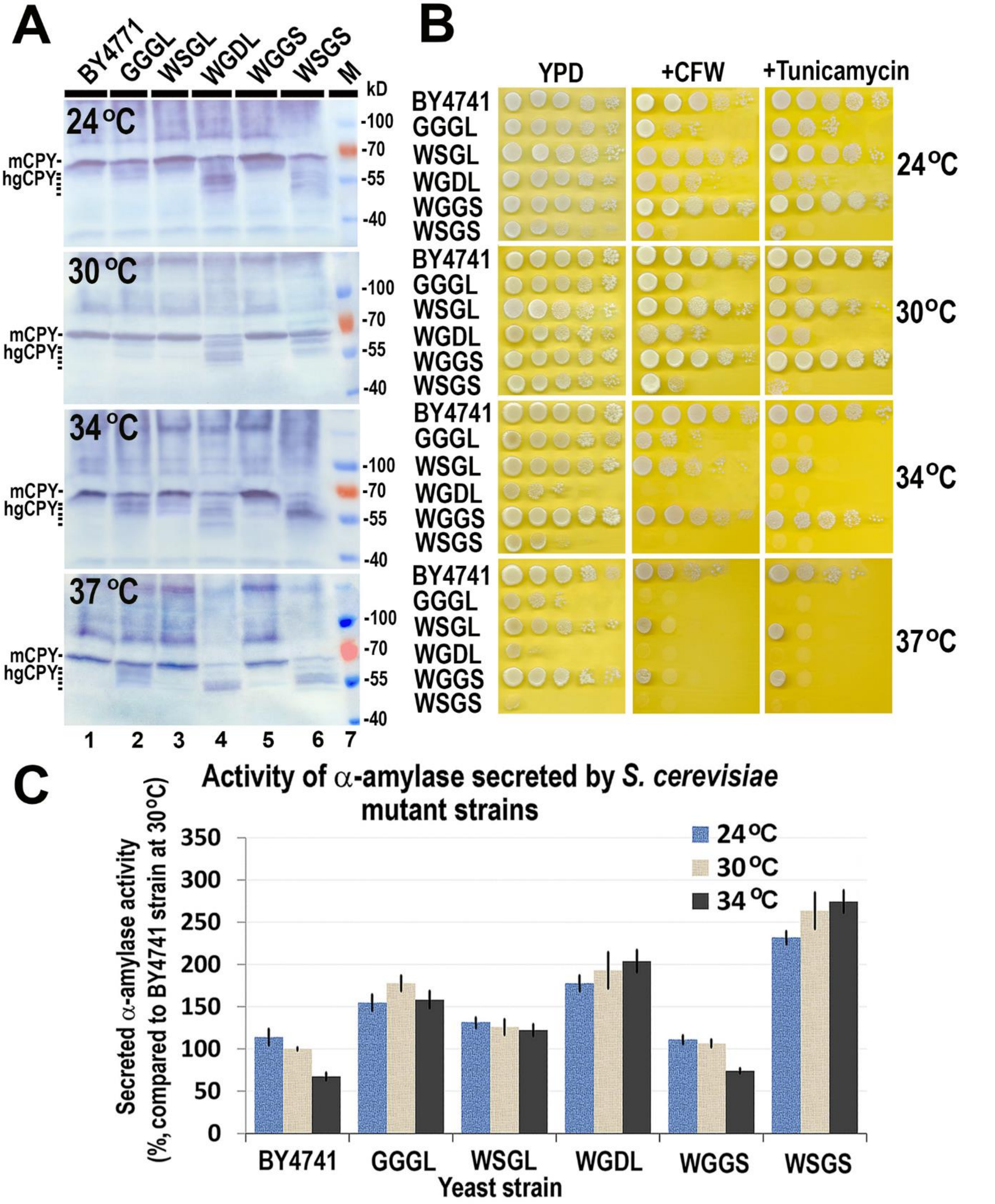

Fig S1-1. Phenotypic characterization of the *S. cerevisiae SEC59* mutants (**A**) Western blot analysis showing the glycosylation status of CPY in the *S. cerevisiae* BY4741 *KlSEC59* mutant

cell lysates. The positions of the mature form of CPY (mCPY) and of the hypoglycosylated forms (hgCPY) are indicated. M: Protein weight marker (Thermo Fisher Scientific Baltics). Results from one out of four independent experiments are presented. (**B)** Analysis of the *S. cerevisiae SEC59* mutant cell growth in response to drugs and temperature. **(C)** Analysis of α-amylase secretion in the *S. cerevisiae SEC59* mutant strains. For normalization, the absorbance ratio of the *S. cerevisiae* BY4741 strain at 30°C was equated to 100%. Values from four independent experiments are shown as the mean ± standard deviation (SD).

In *S. cerevisiae* protein glycosylation defects are associated with alterations in cell wall structure and glycoprotein composition, leading to increased sensitivity to Calcofluor white (CFW), an agent that interacts with cell wall chitin [3]. We assessed the sensitivity of the generated mutants to CFW. Unlike the *K. lactis* DK mutants [1], all generated *S. cerevisiae* DK mutants exhibited sensitivity to CFW, albeit to varying extents. Both CFW and tunicamycin affected the growth of all mutants at 37°C. However, the WGGS and WSGL strains exhibited no sensitivity to CFW at 24°C or 30°C and only slight sensitivity at 34°C (Fig. S1-1B). In contrast, the WSGS demonstrated sensitivity to CFW at all tested temperatures, as did the GGGL and WGDL strains. The impact of the drug on these strains was less pronounced at lower growth temperatures, 24°C or 30°C (Fig. S1-1B).

The efficacy of α-amylase secretion was examined in several clones of all constructed DK mutant strains (Table S1-1) transformed with the pFX7-Amy plasmid as described in [1]. The pFX7-Amy plasmid was constructed after insertion of the *B. amyloliquefaciens* α-amylase gene amplified from the previously described pBori-AMY plasmid [6] into pFX7 vector [7]. Analysis of α-amylase secretion in the *S. cerevisiae* DK-mutant strains revealed a pattern that mirrored the level of CPY glycosylation deficiency level (Fig S1-1C). The WSGS strain secreted almost three times more protein than the wild-type BY4741 strain. The α-amylase secretion levels in the WGDL and GGGL strains were 2.5 and 1.5 times higher than in WT strain and only slightly increased in the WGGS and WSGL strains (Fig S1 C). The efficiency of α-amylase secretion

showed only slight dependence on the temperature at which cells were grown, specifically, at 34°C, secretion was reduced only in the BY4741 and WGGS strains, but not in the other mutant strains. The α-amylase secretion efficacy was not tested at 37°C due to sensitivity of the WSGS and WGDL strains to this temperature (Fig S1-1B).

Collectively, these findings revealed that both *K. lactis* [1] and *S. cerevisiae* DK mutants exhibited protein glycosylation defects. However, in *S. cerevisiae* differences in CPY glycosylation more sensitively reflected mutant DK activity variations, which also corresponded to changes of α-amylase secretion efficacy or sensitivity to drugs. This easily allowed the lineup of tested mutants according to DK activity from highest to lowest: WT, WGGS, WSGL, GGGL, WGDL, and WSGS. It is possible that the defects of protein glycosylation caused by decreased DK activity affect more vital processes in *S. cerevisiae* than in *K. lactis.* This is likely duo to the defects being related not only to N-linked glycosylation, but also to O-linked glycosylation and GPI anchor synthesis, resulting in alterations of cell wall structure and glycoprotein composition.

Suppression of reduced DK activity in the WSGS strain

The efficacy of α-amylase secretion was examined in several WSGS mutant clones transformed with the pFX7-Amy plasmid. It was observed that a subset of these clones lost the ability of increased secretion. As depicted in Fig S1-2 WSGS-1 and WSDS-3 clones secreted only slightly more α-amylase than BY4741 strain cells at 30°C, while the WSGS-2 and WSGS-4 clones continued to secrete α-amylase 2.5-3 times more efficiently than wild-type yeast cells (Fig S1-2A). The Western blot results of lysates of these clones also revealed that CPY was glycosylated more effectively in WSGS-1 and WSGS-3 clones compared to WSGS-2 and WSGS-4 clones (Fig S1-2B). Suspecting that the decrease of the α-amylase secretion efficacy and the restoration of CPY glycosylation in the tested WSGS clones may be attributed to spontaneous reverse or suppressor mutations in the *SEC59* gene, we conducted PCR

amplification of this DK-encoding gene for all four clones using the SD1 and SR1 primer pair and subsequently sequenced it (S2 Table). The analysis of the sequencing results revealed that, while mutations encoding amino acid changes G407S and L421S remained in the *sec59* gene of all four tested clones, the WSGS-1 and WSGS-3 clones exhibited an additional spontaneous mutation encoding the amino acid change E319K (GAG -> AAG). This suggests that the additional E319K mutation partially suppressed the low activity of DK carrying the G407S and L421S mutations.

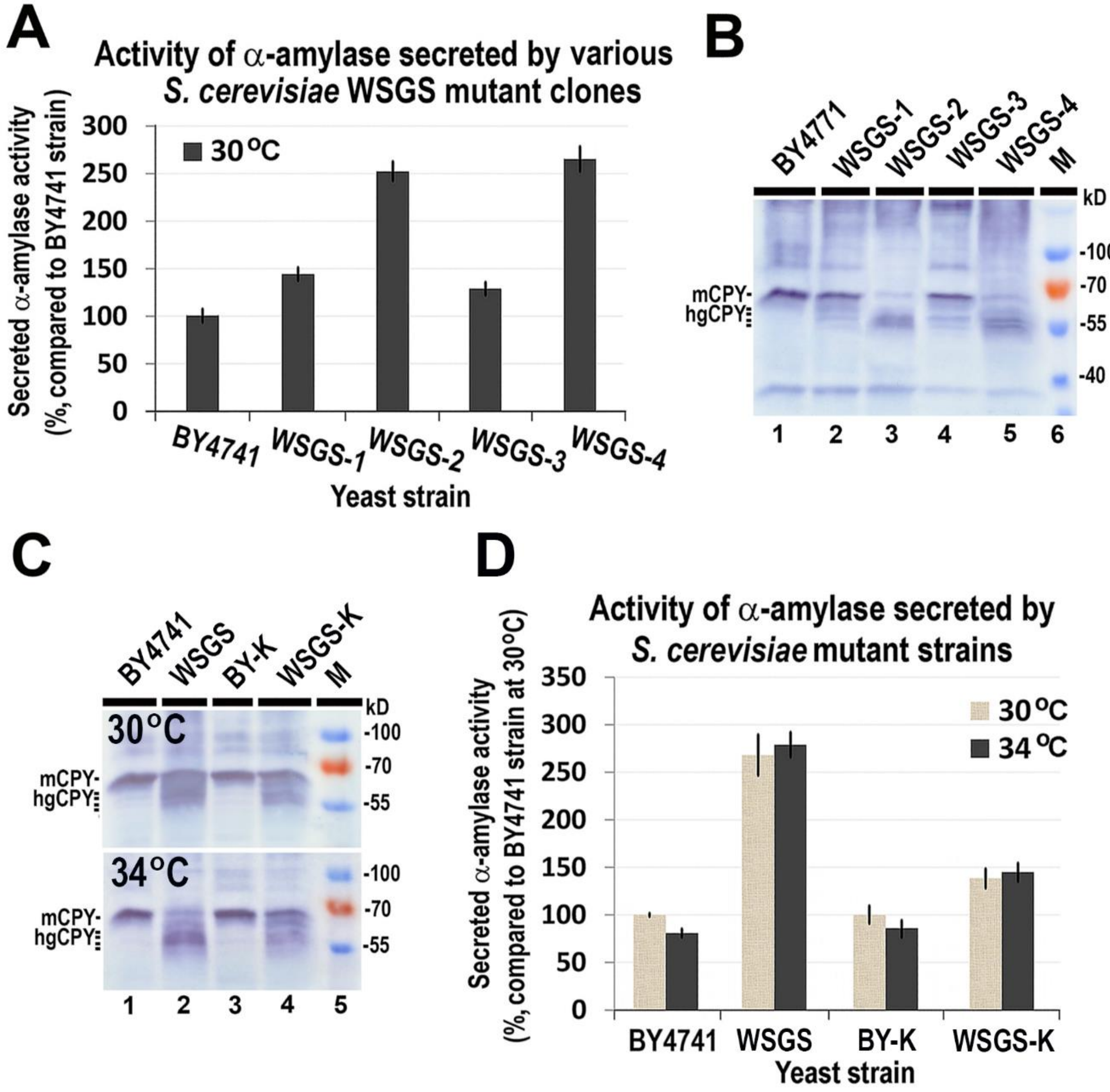

Fig S1-2. Analysis of α-amylase secretion and glycosylation status of CPY in the *S. cerevisiae SEC59* mutants. (**A**) Analysis of α-amylase secretion in in selected WSGS mutant

clones of S. cerevisiae. For normalization, the absorbance ratio of the *S. cerevisiae* BY4741 strain at 30°C was set to 100%. Values from four independent experiments are presented as the mean ± standard deviation (SD); (**B)** Western blot analysis showing the glycosylation status of CPY in cell lysates of selected *S. cerevisiae* WSGS mutant clones; **(C)** Western blot analysis showing the glycosylation status of CPY in cell lysates of the *S. cerevisiae* BY4741, WGSG, BY-K and WSGS-K mutants. The positions of the mature form of CPY (mCPY) and of the hypoglycosylated forms (hgCPY) are indicated. M: Protein weight marker (Thermo Fisher Scientific Baltics). Results from one out of three independent experiments are presented. **(D)** Analysis of α-amylase secretion in the *S. cerevisiae* BY4741, WGSG, BY-K and WSGS-K mutant clones. For normalization, the absorbance ratio of the *S. cerevisiae* BY4741 strain at 30°C was equated to 100%. Values from four independent experiments are presented as the mean ± standard deviation (SD).

To validate this hypothesis, we employed CRISPR-Cas9 technology to introduce the E319K mutation into the DK of BY4741 and WSGS strains. Initially, the mutation encoding the change of DK amino acid E319K was introduced by PCR into pJET-SEC59-1 and pJET-ScSEC59-SS plasmids using the corresponding primers (S2 Table), resulting in the creation of pJET-SEC59-K and pJET-SEC59-SS-K plasmids. These plasmids were then utilized for the amplification of donor DNA. Following the transformation of the wild-type BY4741 strain cells with the pFGgR1-LS3 plasmid, a formaldehyde-resistant transformant was selected and subsequently co-transformed with a mixture including the pKPD-C9 plasmid expressing *S. pyogenes* Cas9 endonuclease gene [1] and marker-free donor DNA fragments. These DNA fragments were PCR amplified either from the pJET-SEC59-K or pJET-SEC59-SS-K plasmids using the SD9 and SR1 primer pair (S2 Table). Transformants were chosen on YNB medium supplemented with formaldehyde. Subsequently, the DK encoding gene fragments from a few selected transformants were PCR-amplified and sequenced. The *S. cerevisiae* strains BY-K and WSGS-K, carrying mutations in the *SEC59* gene encoding amino acid changes E319K (BY-K) and E319K, G407S, and L421S (WSGS-K), were selected. Then we conducted a comparative analysis of CPY glycosylation and α-amylase secretion in the BY4741, WSGS, and newly

constructed BY-K and WSGS-K strains (Table S1-1) to better comprehend the impact of the E319K mutation on DK activity. Intriguingly, DK E319K mutation showed no adverse effects on CPY glycosylation in the BY-K strain, remaining comparable to the parental BY4741 strain. Conversely, the prominently evident CPY glycosylation defects in the WSGS strains were suppressed in the GSGS-K strain and only marginally detectable at both tested temperatures, 30°C and 34°C (Fig S1-2C). As anticipated, the α-amylase secretion pattern in the *S. cerevisiae* BY-K and WSGS-K strains mirrored the level of CPY glycosylation deficiency (Fig S1-2D). The wild-type BY4741 and BY-K strains exhibited comparable protein secretion levels, while the α-amylase secretion in the WSGS strain was nearly three times higher compared to BY4741. Notably, the α-amylase secretion in the WSGS-K strain was reduced to approximately half compared to the WSGS strain, demonstrating a suppressing effect of the introduced E319K mutation (Fig S1-2D).

The amino acid corresponding to the mutated E319K in DK of *S. cerevisiae* in the *K. lactis* DK is 316L, encoded by TTG. The mutation of L (TTG) to K (AAG) requires a two nucleotides change. Given that double transversions are rare events compared to the single transition (GAG to AAG) in *S. cerevisiae*, it is understandable that we haven't observed any suppression in the WSS mutant. Interestingly, the next amino acid in the DK sequence at 320 position is K, which is preserved in the analogous position in the sequence of *K. lactis* DK (317K). It appears that the E319K mutation in *S. cerevisiae* DK results in the doubling of positive charge in this position because of these two lysines. The E319 amino acid is situated in proximity to the DK region (331-346 amino acids), presumed to be responsible for dolichol binding [8]. In spatial terms, it is distant from the G407 and L421 amino acids, which were mutated in the WSGS mutants. The 407 G and 421L amino acids are situated close to the DK motif (430-453 amino acids), which has been identified as the CTP binding domain of the cytidyltransferases [4]. The mechanism by which the E319K mutation suppresses the activity of DK in the WSGS mutant is not yet clear. However, it is possible that the E319K mutation increases the affinity of DK to dolichol.

Consequently, the decrease in the enzyme's activity due to the G407S and L421S mutations in the WSGS mutant may be outweighed by the enhanced binding affinity to dolichol facilitated by the E319K mutation. The G407S and L421S mutations could induce slight alterations in the positions of the protein α-helixes where they are situated, potentially weakening their interaction with the CTP required for the transfer of the phosphate group on dolichol. This, in turn, may lead to a reduction in the activity of the DK. Conversely, the E319K mutation might strengthen this interaction, particularly as it has no negative effect on the activity of the DK in BY-K strain (Fig S1-2C, line 3). Nevertheless, it cannot be ruled out that the helix containing the E319K mutation might approach the helices where the mutated amino acids 407S and 421S are located and through the interactions, it could potentially contribute to the restoration of DK activity. Further investigations involving additional DK mutations are needed to gain a better understanding of the interactions between different DK regions that affects its activity.

References

1. Ziogiene D, Valaviciute M, Norkiene M, Timinskas A, Gedvilaite A. Mutations of *Kluyveromyces lactis* dolichol kinase enhance secretion of recombinant proteins. FEMS Yeast Res. 2019;19(3), pii: foz024.
2. Heller L, Orlean P, Adair WLJr. *Saccharomyces cerevisiae sec59* cells are deficient in dolichol kinase activity. Proc Natl Acad Sci USA. 1992;89:7013-16.
3. Orłowski J, Machula K, Janik A, Zdebska E, Palamarczyk G. Dissecting the role of dolichol in cell wall assembly in the yeast mutants impaired in early glycosylation reactions. Yeast. 2007;24:239-52.
4. Shridas P, Waechter CJ. Human Dolichol Kinase, a Polytopic Endoplasmic Reticulum Membrane Protein with a Cytoplasmically Oriented CTP-Binding Site. J Biol Chem 2006;281:31696–704.

5. Slibinskas R, Samuel D, Gedvilaite A, Staniulis J, Sasnauskas K. Synthesis of the measles virus nucleoprotein in yeast *Pichia pastoris* and *Saccharomyces cerevisiae*. J Biotechnol. 2004;107:115-24.
6. Bartkeviciute D, Sasnauskas K. Studies of yeast *Kluyveromyces lactis* mutations conferring super-secretion of recombinant proteins. Yeast. 2003;20:1–11.
7. Sasnauskas K, Buzaite O, Vogel F, Jandrig B, Razanskas R, Staniulis J, Scherneck S, Krüger DH, Ulrich R. Yeast cells allow the high-level expression and formation of polyomavirus-like particles. Biol. Chem. 1999;380:381-6.
8. Albright CF, Orlean P, Robbins PW. A 13-amino acid peptide in three yeast glycosyltransferases may be involved in dolichol recognition. Proc Natl Acad Sci USA. 1989;19:7366-9.
