# Supplementary material for: Dolichol kinases from yeast, nematode and human can replace each other and exchange their domains creating active chimeric enzymes in yeast
Source: PLoS One. 2024 Nov 7;19(11):e0313330. doi: 10.1371/journal.pone.0313330 (PMC11542857; doi:10.1371/journal.pone.0313330)
Supplement: S2 File — (PDF) [file pone.0313330.s005.pdf]

S2 File

Analysis of protein glycosylation profile in the *K. lactis* WSS mutant using a two-dimensional gel electrophoresis

The depletion of Dol-P in the ER typically leads to protein hypoglycosylation and the activity of DK, which affects the level of Dol-P in the ER, may indirectly influence the regulation of protein glycosylation [1-3]. However, it remains unclear whether the reduced DK activity in yeast DK mutants equally affects the glycosylation of all proteins or if only selected proteins experience significant hypoglycosylation. Since not all yeast proteins are glycosylated, we purified glycosylated proteins from yeast cell lysates of the *K. lactis* WGI-WT strain and WSS mutant using Concanavalin A-Sepharose (ConA-Sepharose), followed by separation using a two-dimensional gel electrophoresis (2DE) system (Fig S2-1). This procedure enriched glycosylated proteins in our samples, ensuring that the spots of separated proteins on 2DE gels were easily detectable. Surprisingly, a comparison of the protein spots in both gels revealed that the amounts of most glycosylated proteins in both *K. lactis* strains, WT-WGI and WSS, remained similar or changed only slightly (Fig S2-1). This suggests that in the case of Dol-P deficiency, the cell tightly regulates the process of protein glycosylation, likely prioritizing the selective restraint of glycosylation for nonessential proteins. To confirm this, we chose five random spots of varying intensity in both gels (refer to Fig S2-1, marked with arrows) and cut the proteins contained in these dots from the gel. The proteins excised from gels were identified at the center of proteomics in Vilnius University Life Science Centre using LC-MSE (Data-Independent Acquisition)-Based Protein Identification method [4].

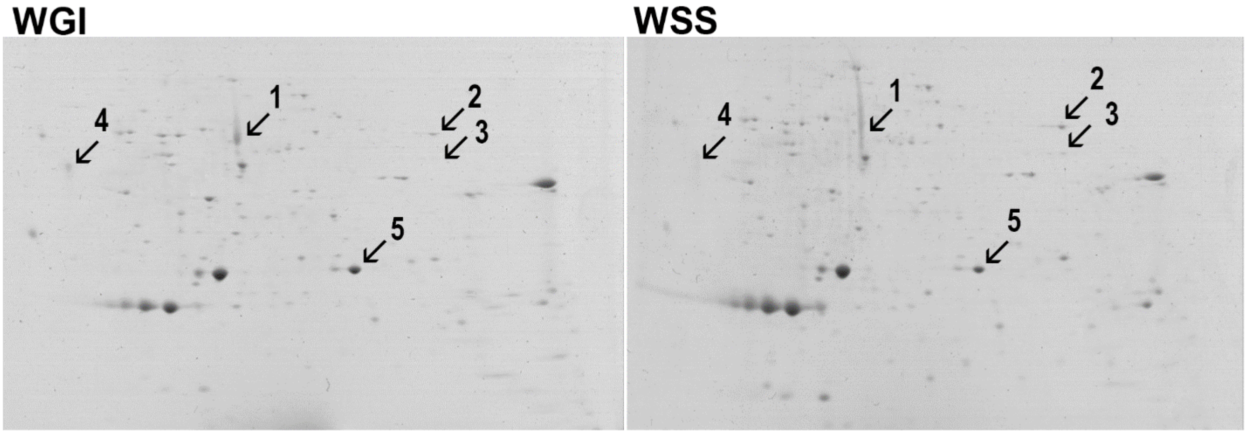

Fig S2-1. Analysis of glycosylated proteins purified with ConA-Sepharose from yeast cell lysates of *K. lactis* WGI strain and WSS mutant, separated by a 2DE system. Five randomly selected spots representing proteins selected for the identification are marked by arrows.

Four out of the five randomly selected glycosylated proteins, whose quantities differed to some extent, likely due to changes in glycosylation in the WSS mutant compared to the WGI strain, were identified as various peptidases (amino-, metallo-, or carboxy-) and two of them were localized in the vacuole (Table S2-1). It is interesting to note that the quantity of the putative metallopeptidase, most likely localized in the cytoplasm (Table S2-1), increased in WSS mutant compared to the WGI strain. However, this increase most likely reflects the appearance of hypoglycosylated forms of the putative metallopeptidase in the WSS mutant rather than the increase of the overall quantity of this protein. Our assumption is supported by the fact that this putative metallopeptidase was identified in the two spots (Fig S2-1, spot numbers 2-3) which represent differently glycosylated proteins isoforms. On the other hand, the appearance of many hypoglycosylated forms of the putative metallopeptidase under conditions of impaired glycosylation is likely due to as many as five predicted glycosylation sites in this protein. The fifth identified protein, whose glycosylation was affected in the WSS mutant, represents one of

the few yeast glyceraldehyde-3-phosphate dehydrogenases (GAPDH) involved in glycolysis, suggesting potential changes in this process under hypoglycosylation conditions.

Table S2-1. Description of the identified randomly selected glycosylated proteins, that exhibited changes in glycosylation in the *K. lactis* WSS mutant.

**Spot Number (Fig. 1)**	**Accession No of identified protein**	**Protein function in *K. lactis***	**Glycosylation changes in WSS strain**	***S. cerevisiae* homolog of identified *K. lactis* protein and its function**
1	Q6CRU2_KLULA	Peptide hydrolase Aminopeptidase, (automatic annotation)	down	Ape3p (AJP84957.1; YBR286W) vacuolar aminopeptidase Y involved in protein catabolism
2-3	Q6CLD9_KLULA	unknown	up	Putative metalloendopeptidase (YIL108W) localized in the cytoplasm in large scale studies which biological role is unknown
4	Q6CXA3_KLULA	Serine-type carboxypeptidase activity	down	CPY (NP_014026.1; PRC1 YMR297W) - serine-type carboxypeptidase involved in vacuolar protein catabolism, vacuolar zymogen activation, phytochelatin biosynthesis and macroautophagy
5	P17819 (G3P1_KLULA)	Glyceraldehyde-3-phosphate dehydrogenase 1	down	AJV45287.1 (Tdh2p YJR009C) - GAPDH, isozyme 2; detected in cytoplasm and cell wall and involved in glycolysis and gluconeogenesis

The results of our investigation of the *K. lactis* glycoproteome revealed that the reduced DK activity in *K. lactis* WSS mutant selectively affected protein glycosylation. The quantities of the

most of the glycosylated proteins did not change significantly and only a minority of glycoproteins in the WSS mutant showed more or less significant changes in glycosylation compared to the WGI strain. This suggest that yeast cells exhibit some tolerance to Dol-P deficiency through adjustments in protein glycosylation. It appears that the disruption caused by Dol-P deficiency is primarily managed by reducing the glycosylation of non-essential, abundant proteins, such as certain vacuolar proteases, while the glycosylation profile of other proteins changes only marginally. It should be kept in mind that in most cases the changes which we see in the gel can be related to the appearance and disappearance of differently glycosylated forms of the respective protein because of underglycosylation. In this case we see more or less variants of the same underglycosylated protein depending upon how many glycosylation sites this protein has. Some spots might disappear at all because the proteins with single glycosylation site after losing glycosylation are not purified by ConA and might be lost. Nevertheless, none of the five identified proteins that underwent changes in glycosylation in the WSS mutant was found to be essential for survival of yeast cells.

Materials and Methods

Purification of glycosylated proteins

Starter cultures of *K. lactis* WGI and WSS strains were grown in YPD medium (20 mL) with shaking at 30°C for 8 hours. These cultures were then used to inoculate 200 mL of fresh YPD medium in new shake-flasks to an initial OD_600 of 0.05, and incubated with agitation at 30°C for 16-18 hours. Cells were collected by centrifugation at 800× g for 5 min, washed with distilled water, weighed, and frozen at -20°C, yielding approximately 3-4 grams of cells per flask.

For cell lysis and protein extraction the thawed cell pellets were resuspended in a Binding buffer (20 mM Tris-HCl, 500 mM NaCl, pH 7.4) with 2 mM PMSF according a v/w formula: 3 mL buffer per gram of cells. Cell disruption was achieved by vortexing with glass beads (twice the cell

pellet weight) for 18 cycles of 30 seconds each, interspersed with 30 seconds of cooling on ice. The cell debris was removed by centrifugation at 800× g for 5 min at 4°C.

For glycosylated protein purification the clarified lysates (~12 mL) were incubated with 300 µL pre-equilibrated in Binding buffer ConA-Sepharose beads (GE Healthcare) in 15 mL centrifuge tubes at 4°C with gentle agitation for 1 hour. Beads were then collected by centrifugation at 1000× g for 2 min at 4°C, and the supernatant was aspirated. Beads were washed with 14 mL of Binding buffer with inversion, followed by centrifugation under the same conditions and removal of the supernatant. During the second wash, the beads were incubated with Binding buffer for 30 min with gentle agitation at 4°C, then again centrifuged and the supernatant was discarded. Glycoproteins were eluted by resuspending the beads in 300 µL of Elution buffer (20 mM Tris-HCl, 500 mM NaCl, 500 mM methyl α-D-glucopyranoside, pH 7.4) and incubating for 10 min on ice with gentle swirling. The supernatant was collected after centrifugation. A second elution step was performed with 600 µL of Elution buffer, and the eluate was combined with the first fraction.

Sample preparation for 2DE

Eluted glycoprotein fractions were concentrated to ~100 µL using 5 kDa cutoff Polyethersulfone (PES) centrifuge concentration columns (Sartorius Stedim Lab Ltd, Stonehouse, UK) by centrifugation at 12000× g, 4°C. After concentration, the samples were precipitated using the conventional TCA/acetone protein precipitation method. One part of the concentrated protein sample was mixed with eight parts of ice-cold acetone, vortexed and then one part of 20% TCA was added to the mixture, vortexed again and incubated for 1h at -20°C. After the incubation, the samples were centrifuged at 15,000× g for 15 min at 4°C, 0.5 mL of ice-cold acetone containing 20 mM DTT was added, and the mixture was centrifuged again at 15,000× g for 15 min at 4°C. The supernatant was aspirated and discarded; the pellets were air-dried. The

precipitated proteins were dissolved in 20 µL of denaturing IEF buffer (7 M urea, 2 M thiourea, 2% CHAPS detergent, 1% ampholytes (pH 3–10, Pharmalyte, GE Healthcare), 0.002% Bromophenol Blue and 75 mM DTT (added just before use) by incubating ~30 min at room temperature and a few vortex/spin cycles. Protein concentrations were determined using a modified Bradford's protein assay (Roti-Nanoquant, Carl Roth GmbH). The protein concentrations were equalized by diluting samples with IEF buffer. Prepared samples were stored frozen at -80°C until use.

Non-equillibrium pH Gel Electrophoresis-based first-dimension and SDS-PAGE second-dimension protein separations and preparation of proteins excised from gels

All the first-dimension separation procedures, in-house gel solution recipes, gel casting and running conditions were made identically as described by Zinkevičiūtė et al. [5]. In this experiment, we loaded 30 µg of purified glycosylated protein sample per gel. The second dimension running conditions, fixing, and staining of the gels were performed identically as described by Slibinskas et al. [6].

Samples of proteins excised from gels were prepared as described by Shevchenko et al. [7].

References

1. Schenk B, Fernandez F, Waechter CJ. The ins(ide) and out(side) of dolichyl phosphate biosynthesis and recycling in the endoplasmic reticulum. Glycobiology. 2001;11: 61R-70R.
2. Fernandez F, Shridas P, Jiang S, Aebi M, Waechter CJ. Expression and characterization of a human cDNA that complements the temperature-sensitive defect in dolichol kinase activity in the yeast sec59-1 mutant: the enzymatic phosphorylation of dolichol and diacylglycerol are catalyzed by separate CTP-mediated kinase activities in Saccharomyces cerevisiae.

3. Denecke J, Kranz C. Hypoglycosylation due to dolichol metabolism defects. Biochim Biophys Acta. 2009; 1792(9):888-95.
4. Distler U, Kuharev J, Navarro P, Levin Y, Schild H, Tenzer S. Drift time-specific collision energies enable deep-coverage data-independent acquisition proteomics. Nat Methods. 2013;11: 167–70.
5. Zinkevičiūtė R, Ražanskas R, Kaupinis A, Macijauskaitė N, Čiplys E, Houen G, et al. Yeast Secretes High Amounts of Human Calreticulin without Cellular Stress. Curr Issues Mol Biol. 2022;44: 1768–87.
6. Slibinskas R, Ražanskas R, Zinkevičiūtė R, Čiplys E. Comparison of first dimension IPG and NEPHGE techniques in two-dimensional gel electrophoresis experiment with cytosolic unfolded protein response in Saccharomyces cerevisiae. Proteome Sci. 2013;11(1): 36.
7. Shevchenko A, Tomas H, Havlis J, Olsen J V, Mann M. In-gel digestion for mass spectrometric characterization of proteins and proteomes. Nat Protoc. 2006;1: 2856–60.
