# Supplementary figures and images for: Dolichol kinases from yeast, nematode and human can replace each other and exchange their domains creating active chimeric enzymes in yeast
Source: PLoS One. 2024 Nov 7;19(11):e0313330. doi: 10.1371/journal.pone.0313330 (PMC11542857; doi:10.1371/journal.pone.0313330)

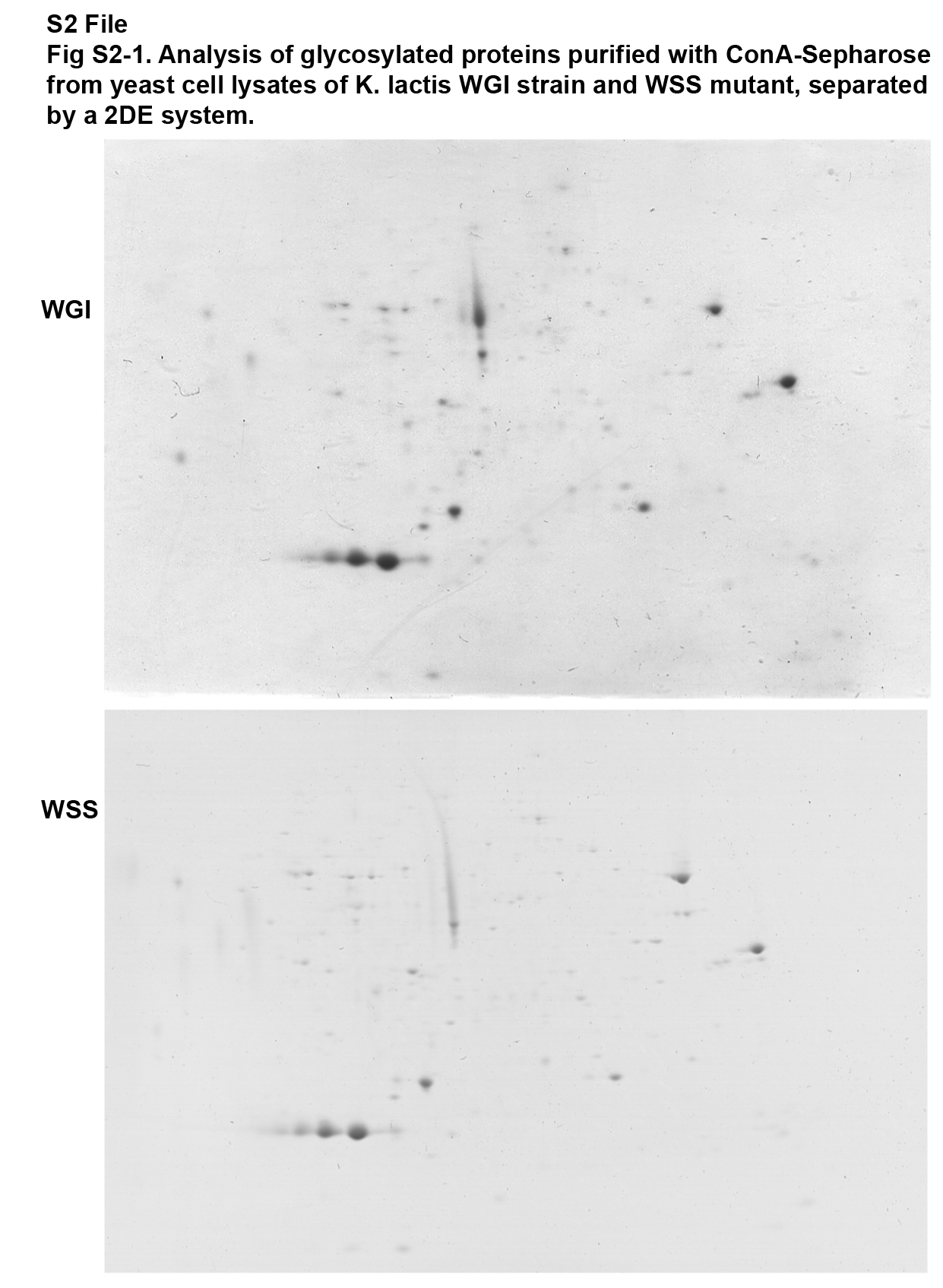

Supplement: S1 Raw images — (TIF) [file pone.0313330.s006.tif]

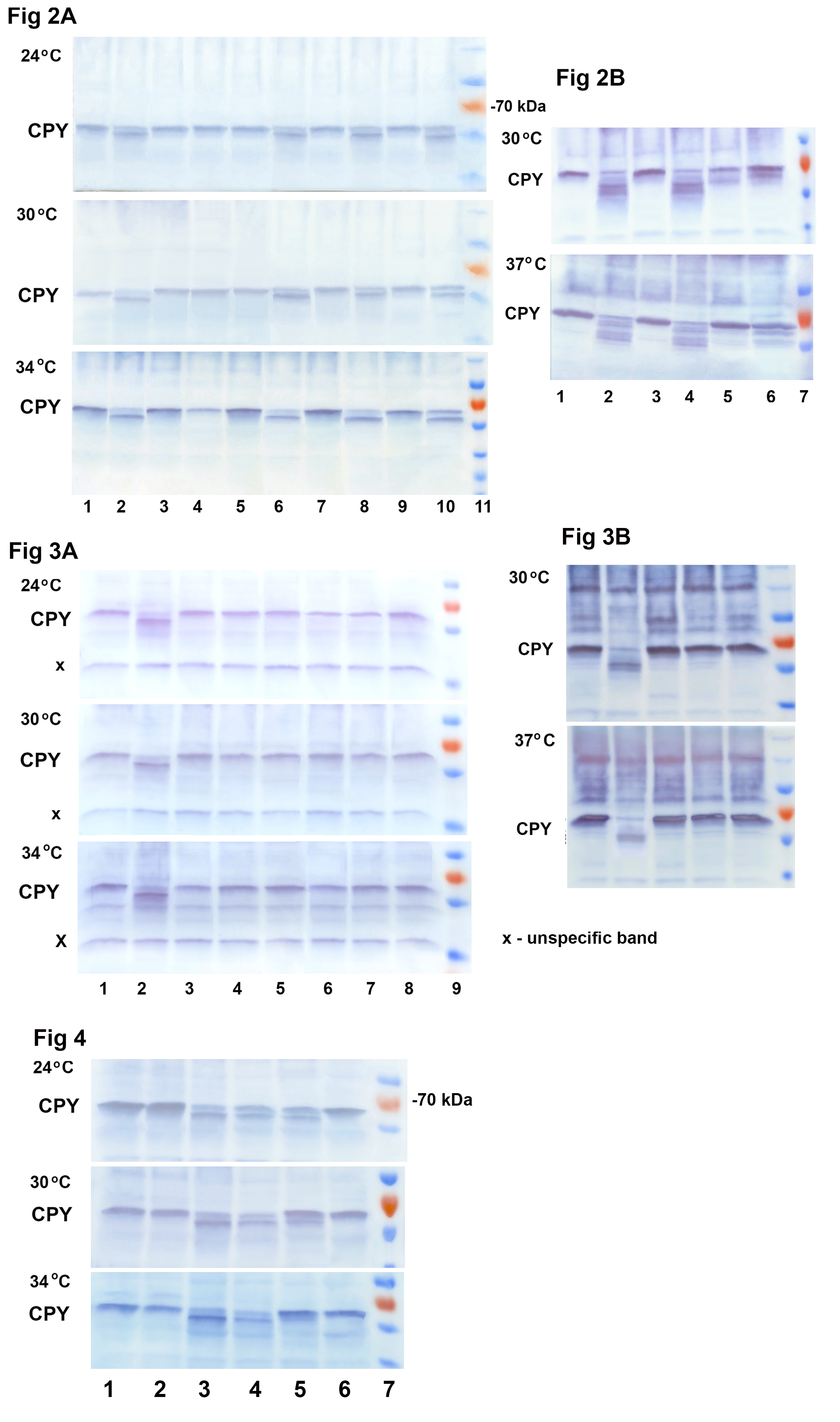

Supplement: S2 Raw images — (TIF) [file pone.0313330.s007.tif]
